# Cloning and Functional Analysis of Glyoxalase I Gene *BrGLYI 13* in *Brassica rapa* L.

**DOI:** 10.3390/ijms26062737

**Published:** 2025-03-18

**Authors:** Xiaojia Song, Feng Zhang, Xiaolei Tao, Yapeng Li, Tingting Fan, Junyan Wu, Li Ma, Lijun Liu, Yuanyuan Pu, Wangtian Wang, Gang Yang, Wancang Sun

**Affiliations:** 1 College of Agronomy, Gansu Agricultural University, Lanzhou 730070, China; songxiaojia0128@163.com (X.S.); 1258220089zf@gmail.com (F.Z.); txl162185@163.com (X.T.); 15379222259@163.com (Y.L.); fantt@gsau.edu.cn (T.F.); wujuny@gsau.edu.cn (J.W.); puyy@gsau.edu.cn (Y.P.); 2 State Key Laboratory of Arid Land Crop Science, Lanzhou 730070, China; mal@gsau.edu.cn (L.M.); liulj@gsau.edu.cn (L.L.); 3 College of Life Science and Technology, Gansu Agricultural University, Lanzhou 730070, China; wtwang@gsau.edu.cn

**Keywords:** *Brassica rapa* L., genome-wide identification, *BrGLYI13*, self-compatibility, functional analysis

## Abstract

Glyoxalase I (GLYI) is a key enzyme that detoxifies methylglyoxal, a toxic byproduct of glycolysis, and is essential for plant pollination. However, the genome-wide identification and functional analysis of *GLYI* in *Brassica rapa* L. (*B. rapa*) remain limited. This study identified 17 *BrGLYI* genes (*BrGLYI1*–*BrGLYI17*) from the *B. rapa* genome. The self-compatible line 039-1 and the self-incompatible line GAU-28-5 were used as experimental materials, and Real-Time Quantitative Reverse Transcription PCR (RT-qPCR) was performed to examine the effect of *BrGLYI* genes on self-compatibility in winter *B. rapa*. Preliminary results showed that *BrGLYI13* exhibited significant tissue specificity, with higher expression in the flowers of 039-1 compared to GAU-28-5. The open reading frame of *BrGLYI13* (852 bp) was cloned from both 039-1 and GAU-28-5 cDNA, with no base mutations observed between the two lines. RT-qPCR revealed higher *BrGLYI13* expression in the stigma of 039-1 compared to GAU-28-5. Based on the functional conservation and sequence homology, *BrGLYI13* is speculated to play a similar role to that of *AtGLYI3* in methylglyoxal detoxification and stress response. Furthermore, the knockout of *AtGLYI3* resulted in reduced silique lengths and seed numbers. These findings suggest that *BrGLYI13* is involved in the self-compatibility response in *B. rapa* and promotes the silique length and seed number in the Arabidopsis mutant, providing a basis for further research on the mechanisms of self-compatibility in *B. rapa*.

## 1. Introduction

Plants accumulate excessive toxic substances, such as methylglyoxal (MG), under stress conditions [1]. At low concentrations, MG can function as a signaling molecule that activates the plant’s self-protection mechanisms. However, when MG levels increase to harmful concentrations, it can damage critical cellular structures, including DNA, RNA, proteins, and biological membranes, leading to loss of function and adversely affecting the plant’s normal growth and development [2,3]. Therefore, maintaining a dynamic balance of MG levels is crucial for plant growth and development. To cope with this, plants have evolved the glyoxalase system, which detoxifies MG by converting it into harmless compounds [4]. This pathway involves a complex regulatory network composed mainly of three enzymes: glyoxalase I (GLYI), glyoxalase II (GLYII), and glyoxalase III (GLYIII) [5]. The MG detoxification mechanism can be divided into two main categories: glutathione (GSH)-dependent and GSH-independent systems [6,7]. When GSH is present, MG first reacts with GSH to form a thiohemiketal compound, which is then converted into S-D-lactoylglutathione (SLG) under the catalytic action of GLYI. Subsequently, SLG is further degraded into harmless D-lactic acid and reduced GSH through the action of GLYII. In the absence of GSH, MG is directly converted into non-toxic D-lactic acid by GLYIII [8]. This series of enzyme-catalyzed reactions effectively mitigates the potential damage MG could cause to the cell.

As the first key enzyme in the GSH-dependent MG detoxification pathway, GLYI plays a critical role. However, our understanding of glyoxalase enzymes in plants is still limited compared to other organisms. In recent years, the availability of genome-wide sequencing data has expanded the possibilities for identifying glyoxalase families in plants. Through the evolutionary process, gene amplification and duplication have resulted in the formation of gene families for most plant genes [9]. Xu et al. [10] demonstrated that GLYI belongs to a multigene family in plants, with the number of family members varying across different species. Studies on the *glyoxalase* gene family in various plants, including *Oryza sativa* L., *Arabidopsis thaliana* L. (*A.thaliana*), *Solanum lycopersicum* L., *Triticum aestivum* L., *Saccharum officinarum* L., and Chinese cabbage (*Brassica rapa* L.) [11,12,13,14], have revealed that glyoxalases are involved in a variety of biological processes, including stress responses, seed germination, plant aging, nutrient regulation, signal transduction, starch synthesis, and pollen development [15,16,17]. However, such studies have not yet been reported on winter *B. rapa*, and new roles for glyoxalases in various developmental and signaling pathways are continuing to emerge.

It has been demonstrated that the MG detoxification process mediated by glyoxalases is crucial for pollination responses. In *Brassica* species, during self-incompatibility (SI), MG interacts with affinity factors involved in proteasomal degradation, and the accumulation of glyoxalases facilitates MG detoxification, ensuring effective pollination. Research by Sankaranarayanan et al. shows that glyoxalase I (GLO1) acts as an affinity factor essential for successful pollination [18]. In *Brassica napus* L. (*B. napus*), SI is triggered by an allele-specific interaction between a cysteine-rich small secreted protein (SCR/SP11) encoded by pollen and a stigma receptor kinase (SRK), which activates the Armadillo repeat-containing 1 (ARC1) E3 ubiquitin ligase, leading to the degradation of GLO1 [19]. Furthermore, the downregulation of GLO1 is linked to SI, and its overexpression can break SI [20].

With the successful breeding of cold-tolerant winter *B. rapa* varieties, such as “Longyou 6” and “Longyou 7”, which can withstand extreme cold temperatures as low as −30 °C, alongside the promotion of efficient cultivation techniques, these crops have brought about significant ecological and economic benefits, particularly in northern China. These varieties have become the primary oilseed crops capable of overwintering safely in the region [21]. *B. rapa* is a cross-pollinated crop that exhibits SI [22]. While SI enhances species adaptability and helps maintain genetic diversity, it also leads to inbreeding depression, complicating pure-line breeding, hindering variety improvement, and reducing the effective lifespan of the varieties [23]. Therefore, investigating the mechanisms underlying self-compatibility (SC) and SI in *B. rapa* is of great significance for its future improvement.

Research has shown [24] that *A. thaliana* contains the molecular components necessary for SI signal transduction, making it a valuable model for studying the SI mechanisms in *Brassica* species. Evaluating candidate SI genes and identifying SI signal molecules in *A. thaliana* not only aids in understanding the molecular mechanisms of SI but also provides insights into the evolutionary aspects of the SI mechanisms in *Brassicaceae* plants. While research on SC in *Brassica* species has primarily focused on *A. thaliana* and Chinese cabbage, studies on SC in winter *B. rapa* remain scarce, especially with regard to the signaling pathways of SC genes, which are still largely unexplored.

While numerous experimental studies have confirmed the stress-resistant functions of *GLYI* genes in plants, the role of *GLYI* genes in *B. rapa* remains unexplored. Therefore, this study aimed to systematically identify and analyze the *BrGLYI* gene family in the *B. rapa* genome. Using both self-compatible and self-incompatible lines of *B. rapa* as experimental materials, molecular biology techniques were employed to clone the *BrGLYI13* gene. Subsequently, bioinformatics analysis was performed to investigate its tissue-specific expression characteristics. Functional analysis was conducted using *A. thaliana* mutants to explore the relationship between this gene and SC, providing valuable insights into the role of *BrGLYI13* in the SC mechanism of winter *B. rapa*.

## 2. Results

### 2.1. Identification and Analysis of BrGLYI Gene Family Members

Using BLAST 2.14.0 (NCBI, 2023) and HMMER 3.4 (Eddy, 2022) searches, redundant sequences were removed, and gene family members were confirmed through domain analysis. A total of 17 *GLYI* gene family members were identified in the *B. rapa* genome. These members were named *BrGLYI1* to *BrGLYI17* based on their chromosomal distribution (Table 1). Proteins containing the glyoxalase (PF00903) conserved protein domain with lactoylglutathione lyase activity were classified as GLYI proteins. We summarized the physicochemical properties of all *GLYI* family members in *B. rapa*, including the gene ID, amino acid count, isoelectric point (pI), and molecular weight (MW), and predicted the subcellular localization.

The results indicated that the molecular weights of the proteins encoded by this gene family ranged from 12,967.91 Da to 115,660.97 Da, with theoretical isoelectric points varying from 4.84 (*BrGLYI10*) to 8.85 (*BrGLYI11*). The aliphatic index ranged from 69.74 to 100.76, suggesting a high proportion of aliphatic amino acids in the proteins, all of which were predicted to be hydrophobic. Hydrophilicity/hydrophobicity predictions revealed that most of the proteins were hydrophilic, with the exception of *BrGLYI16*, which was hydrophobic. The hydrophilic proteins were found to be 16 times more hydrophilic than the hydrophobic proteins. Instability index predictions indicated that 7 proteins were stable in vitro, while 10 proteins had an instability index greater than 40. Subcellular localization predictions showed that 11 GLYI proteins were located in the cytoplasm, 3 in the chloroplasts, and 3 in the nucleus. These variations in subcellular localization suggest functional diversity and evolutionary divergence within the gene family.

### 2.2. Phylogenetic Analysis and Classification of the BrGLYI Gene Family

**Figure 1:**
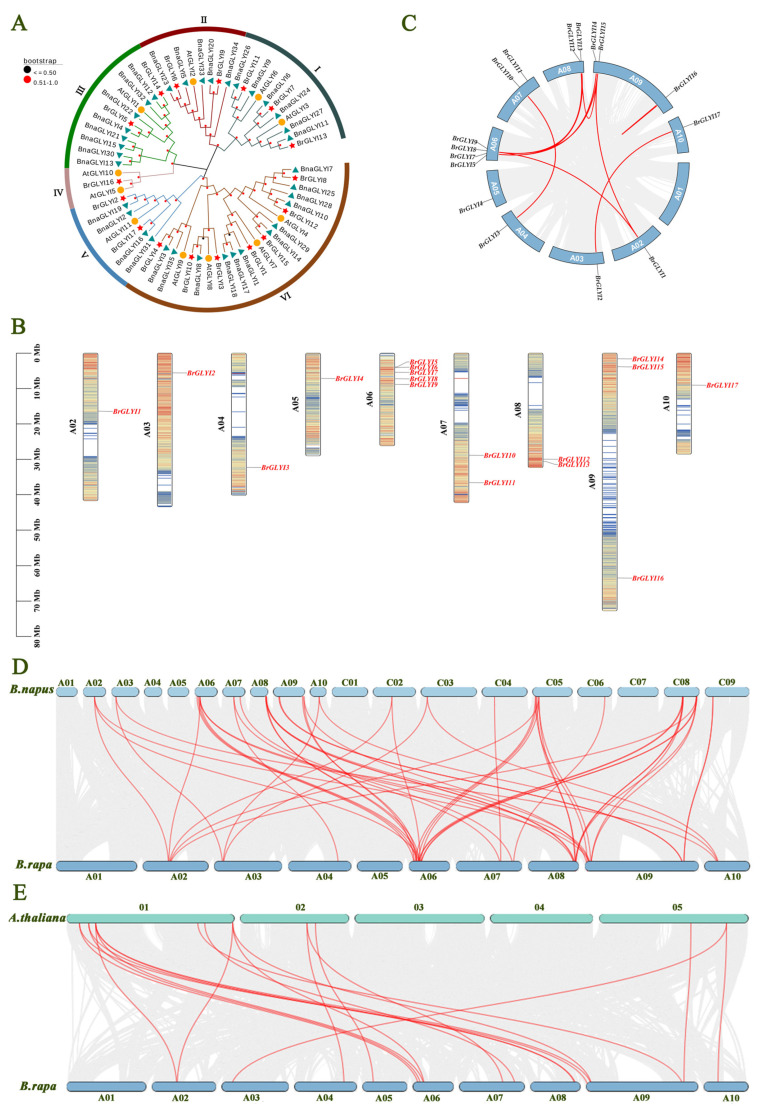
Genome-wide identification and analysis of *BrGLYI* gene family in winter *B. rapa*: (**A**) The phylogenetic relationships of *BrGLYΙ* across different plant species were analyzed through the construction of a phylogenetic tree. This tree was generated using the Neighbor-Joining method in MEGA11, based on multiple sequence alignments. Bootstrap values, obtained from 1000 replicates, are displayed above the branches to indicate support levels. A total of 63 GLYI proteins, including *B. napus* (35), *B. rapa* (17), and *A. thaliana* (11), were included in the phylogenetic analysis of *BrGLYΙ*. Subfamilies I to VI are color-coded. “*At*”, “*Bna*”, and “*Br*” refer to the proteins in *A. thaliana*, *B. napa*, and *B. rapa*. Additionally, proteins from these species are marked with specific symbols: yellow circles for *A. thaliana*, green triangles for *B. napus*, and red stars for *B. rapa*. The sequences utilized in this analysis are provided in the Supplementary File. (**B**) The chromosomal distribution of *GLYI* genes in *B. rapa*. (**C**) Analysis of homology and collinearity among members of the *BrGLYI* gene family. (**D**) Comparative analysis of homology and collinearity between *BrGLYI* and *BnaGLYI*. (**E**) Comparative analysis of homology and collinearity between *BrGLYI* and *AtGLYI*.

To better understand the evolutionary relationships and functions of BrGLYI proteins, we constructed a phylogenetic tree using the sequences of 17 *B. rapa* GLYI proteins, 11 *A. thaliana* GLYI proteins, and 35 *B. napus* GLYI proteins (Figure 1A). Phylogenetic analysis revealed that the 17 GLYI proteins clustered into six distinct groups (Group I to Group VI). These *GLYI* genes were distributed across nearly all branches of the tree, with Group VI containing the largest number of members—7 from *B. rapa*, 4 from *A. thaliana*, and 12 from *B. napus*. The genetic differentiation of *GLYI* genes was observed between these species and winter *B. rapa*. Further analysis of the clustering patterns showed that sequences with high similarity grouped together, indicating that these genes likely evolved from a common ancestor and may share similar or identical functions.

To visually explore the distribution of *GLYI* genes on the chromosomes of *B. rapa*, we mapped all identified family members to the chromosomes (Figure 1B). The results showed that the 17 *GLYI* genes were randomly distributed across nine chromosomes, with 5 genes located on chromosome A06, 3 on chromosome A09, and the remaining genes scattered across chromosomes A02, A03, A04, A05, A07, A08, and A10. The distribution of *GLYI* genes across the chromosomes was uneven, and tandem gene arrangements were observed, which are thought to play a key role in gene duplication and recombination.

To investigate the evolutionary relationships of the *BrGLYI* gene family in *B. rapa*, we conducted an intraspecies synteny analysis (Figure 1C). The analysis revealed that the 17 *GLYI* genes in the family exhibited 10 pairs of synteny relationships. The highest number of syntenic genes was found between chromosomes A02, A06, A08, and A09. Additionally, some synteny pairs involved multiple genes corresponding to a single gene, suggesting gene duplication events within the family. These duplication events have likely played a significant role in the evolutionary development of *B. rapa*.

The interspecies synteny analysis of the *GLYI* gene family between *B. rapa* and *B. napus* (Figure 1D) revealed that, except for chromosomes A01 and A05 in *B. rapa*, the remaining eight chromosomes shared 52 pairs of homologous genes with *B. napus*, with the highest number of homologous gene pairs found on chromosome A06. An interspecies synteny analysis between *B. rapa* and *A. thaliana* (Figure 1E) identified synteny relationships on chromosomes 1, 2, and 5 of *A. thaliana*, with 19 homologous gene pairs observed. The highest number of syntenic gene pairs (13 pairs) was found between *BrGLYI* genes and chromosome 1 of *A. thaliana*.

### 2.3. Expression Analysis of BrGLYIs in B. rapa

**Figure 2:**
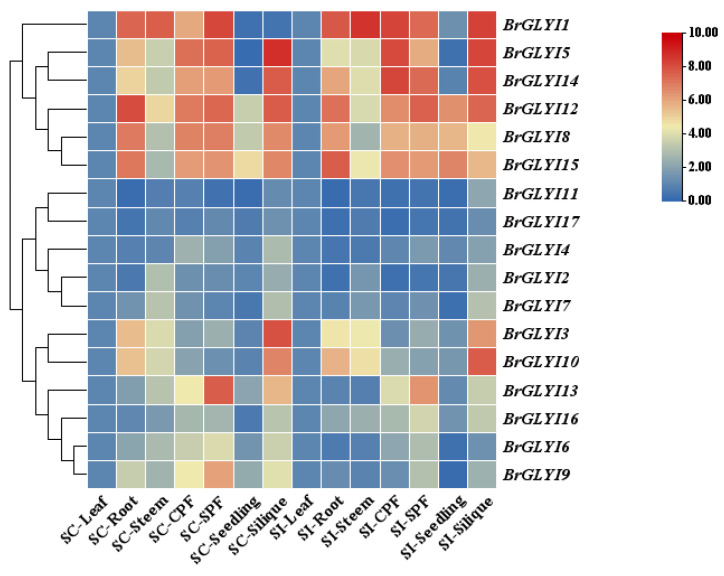
Expression of *BrGLYI* gene family in different tissues. SC: self-compatibility material; SI: self-incompatibility material; CPF: cross-pollinated flowers; SPF: self-pollinated flowers. Relative gene expression was calculated using the comparative 2^−ΔΔCT^ method. All data were standardized using actin as an internal reference gene. Three independent biological replicates were performed. The color scale indicates the log2-transformed average relative expression levels from the three replicates, with data normalized to the expression levels in leaf tissue.

To gain deeper insights into the biological function of the *BrGLYI* gene family in *B. rapa*, this study examined the expression patterns of *BrGLYI* genes across various tissues in the self-compatible line 039-1 and the self-incompatible line GAU-028-5. The results are shown in Figure 2. The expression level in the leaves was used as the control, with red indicating relatively high gene expression and blue indicating relatively low expression. The expression of the 17 *BrGLYI* genes showed significant variation across different tissues of *B. rapa*. Among these, *BrGLYI1*, *BrGLYI5*, *BrGLYI14*, *BrGLYI12*, *BrGLYI8*, and *BrGLYI15* were highly expressed in multiple tissues. Notably, the expression of *BrGLYI13* was particularly high in the flowers of the self-compatible line 039-1 but significantly lower in GAU-028-5. Additionally, its expression in the self-compatible pods was higher than that in the self-incompatible pods. These findings suggest that *BrGLYI13* may play a positive regulatory role in the SC of *B. rapa*. Therefore, further functional studies on this gene are warranted.

### 2.4. Cloning and Sequence Alignment of BrGLYI13

**Figure 3:**
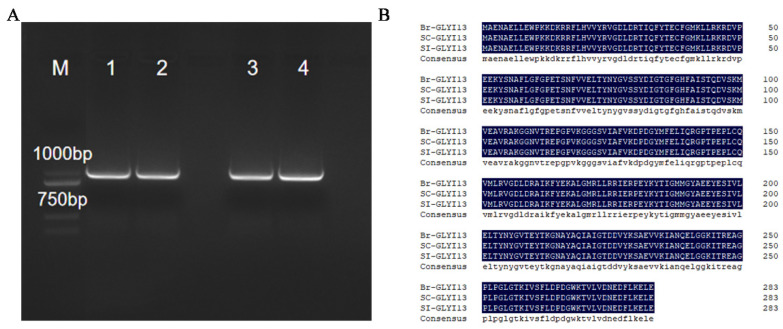
(**A**) Identification of *BrGLYI13* gene-positive clones. Note: M: DL2000; Lanes 1, 2: PCR amplification products of self-compatible lines 039-1; Lanes 3, 4: PCR amplification products of self-incompatible lines GAU-28-5. “M” represents the molecular weight marker (Marker). (**B**) Results of *BrGLYI13*-encoded protein sequence alignment.

The cDNA templates obtained from unpollinated and pollinated stigmas of both the self-compatible and self-incompatible lines were used for the PCR amplification. Gel electrophoresis of the PCR products revealed bands with sizes ranging from approximately 750 to 1000 bp (Figure 3A). PCR screening of positive recombinant clones similarly identified a target band around 1 kbp. Sequencing analysis of the positive clones confirmed that the CDS of the target gene was 852 bp in length (Figure S1). The amplification results were consistent with the predicted size of the CDS fragment, thus confirming the successful cloning of the *BrGLYI13* gene.

The CDS region sequence of the *BrGLYI13* gene cloned from two strains was aligned with the CDS region sequence of the *BrGLYI13* gene in the winter *B. rapa* genome using DNAMAN software 9.0 (Figure S1), which shows a high degree of consistency in the DNA sequence. A comparison of the protein sequences encoded by the gene (Figure 3B) revealed a 100% consistency in the amino acid sequence. This collectively indicates a high level of sequence conservation for the gene.

### 2.5. Identification and Analysis of Cis-Acting Elements in the Promoter Region of BrGLYI13

**Figure 4:**
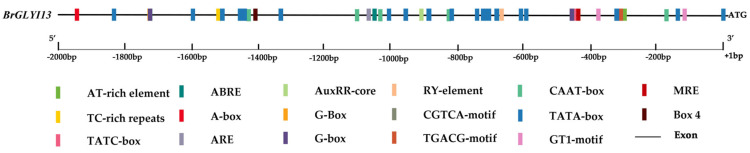
Promoter cis-acting regulatory element distribution. Different-colored rectangular boxes represent individual cis-acting components; ARE—anaerobic induction response element; TGACG—motif–methyl jasmonate response element; G-box—light response element; ABRE—abscisic acid response element; G-Box—optical response element; GT1-motif—optical response element; TC-rich repeats—defense and stress response components; RY-element—seed-specific regulatory elements; MRE—optical response element. Note: +1 is the “A” of the initiating ATG codon.

The *BrGLYI13* gene promoter was analyzed using the PlantCARE website to predict cis-acting elements. As shown in Figure 4, several cis-acting elements involved in the light response, abscisic acid (ABA) response, and MYB transcription factor binding sites were identified within the 2 kbp region upstream of the ATG start codon. The analysis revealed that the *BrGLYI13* gene promoter contains multiple light-responsive elements, as well as a range of other regulatory elements, including those responsive to anaerobic conditions, ABA, low temperature, and methyl jasmonate (Figure 4). These findings suggest that the *BrGLYI13* gene is closely involved in regulating plant growth, development, and responses to various abiotic stresses.

### 2.6. Expression Analysis of BrGLYI13 in Different Tissues of B. rapa

**Figure 5:**
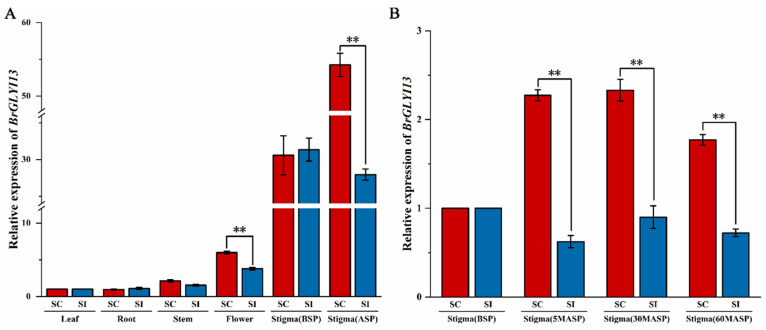
*BrGLYI13* tissue expression specificity. (**A**) Expression levels of *BrGLYI13* in different tissues. BSP: Before Self-Pollination; ASP: After Self-Pollination; the stigma collection was performed 30 min after self-pollination. Relative gene expression was calculated using the comparative 2^−ΔΔCT^ method. All data were normalized to the actin expression level as an internal control, with expression levels in leaf tissue set to 1. Three independent biological replicates were performed. The “**” symbol indicates a significant difference (*p* ≤ 0.05) according to analysis of variance. (**B**) Expression levels of *BrGLYI13* in stigma tissues of *B. rapa* with different compatibility types under different self-pollination times. BSP: Before Self-Pollination; MASP: Minutes After Self-Pollination. Relative gene expression was calculated using the comparative 2^−ΔΔCT^ method. All data were normalized to the actin expression level as an internal control, with expression levels in pre-self-pollinated stigmas set to 1. Three independent biological replicates were performed. The “**” symbol indicates a significant difference (*p* ≤ 0.05) according to analysis of variance.

Since no sequence variation was observed, we hypothesized that the expression levels of *BrGLYI13* might differ between the stigmas of the self-compatible and self-incompatible lines (Figure 5A). Gene expression analysis revealed that *BrGLYI13* was expressed in various tissues of *B. rapa*, with significant differences in its expression levels. In the stigma prior to self-pollination, the gene was expressed in both the self-compatible and self-incompatible lines, with no significant differences in its expression levels, suggesting a basal activity of the gene in the stigma. After self-pollination, the expression level of *BrGLYI13* was the highest in the stigma, with values of 54.24 in the SC line 039-1 and 27.94 in the SI line GAU-28-5, a difference of approximately 1.94 times, which was statistically significant. The expression levels in the flowers were slightly lower, with values of 5.98 in 039-1 and 3.78 in GAU-28-5, a difference of about 1.58-fold, which was also statistically significant. In contrast, the expression levels in the root and stem were significantly lower than those in the stigma and flower, with no significant difference observed between the two lines. These results suggest that *BrGLYI13* is expressed in all tissues of *B. rapa*, but its primary function appears to be in the stigma, where it may play a role in the SC of the stigma.

The RT-qPCR analysis of the *BrGLYI13* expression in the stigma after the self-pollination treatment (Figure 5B) revealed that the *BrGLYI13* expression followed a distinct pattern, characterized by an initial increase followed by a decrease. At 5 min post-pollination, the expression level was significantly higher than that of BSP, and the expression level reached its peak at 30 min post-pollination. The increase in the *BrGLYI13* expression may be linked to the surge in MG levels and the corresponding rise in MG-modified proteins. Previous studies have shown that MG levels rapidly increase within 10 min after self-pollination and return to baseline levels after 60 min [18]. To maintain MG concentrations at non-toxic levels in the stigma, the *BrGLYI13* expression was initially upregulated, which likely explains the subsequent downregulation after the peak.

The graph shows that the relative expression of *BrGLYI13* in the self-compatible line 039-1 reached approximately 2.3-fold at 30 min post-self-pollination, while the relative expression in the self-incompatible line GAU-28-5 was around 0.9, resulting in a difference of approximately 2.6-fold. This time point corresponds to the critical period for the self-incompatibility reaction, where self-pollination at 30 min significantly induces *BrGLYI13* expression in the stigma. These findings suggest that *BrGLYI13* plays a role in the self-compatibility response in *B. rapa* after self-pollination.

### 2.7. Effect of T-DNA Insertion Homozygous Mutant of AtGLYI3 on Self-Compatibility in A. thaliana

#### 2.7.1. Identification of DNA and Transcript Levels in Homozygous Mutants

**Figure 6:**
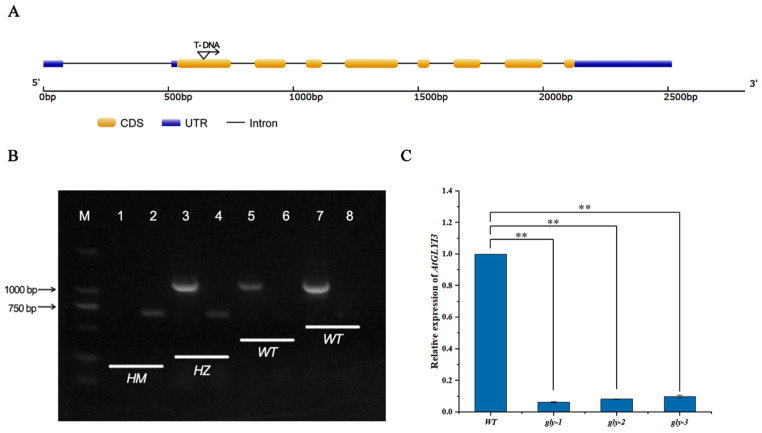
Identification of *gly* homozygous mutants. (**A**) *AtGLYI3* gene structure and T-DNA insertion site. “M” represents the molecular weight marker (Marker). (**B**) Gene-level detection in *gly* mutants: HM represents homozygous *A. thaliana* strain, HZ represents heterozygous *A. thaliana* strain, and WT represents wild-type *A. thaliana* strain. Channels 1, 3, 5, and 7 correspond to LR + PR, while channels 2, 4, 6, and 8 correspond to LB + RP. (**C**) Detection of transcription levels of *gly* mutants. Relative gene expression was calculated using the comparative 2^−ΔΔCT^ method. All data were normalized to the *actin2* expression level. Three independent biological replicates were performed. The “**” symbol represents significant difference (*p* ≤ 0.05) according to analysis of variance.

To further explore the function of *BrGLYI13*, a comparative study on self-compatibility was conducted using the Arabidopsis mutant as the experimental material. The sequence alignment (Figure S2) revealed a high degree of homology between the *A. thaliana AtGLYI3* gene and the *B. rapa BrGLYI13* gene (nucleotide sequence identity = 90.50%; protein sequence identity = 93.66%). As shown in Figure 6A, *AtGLYI3* consists of nine exons and eight introns, with the T-DNA insertion site in the *gly* mutant located within the first exon.

PCR amplification was performed using specific primers (LP and RP) for the *AtGLYI3* gene, with leaf DNA from the *gly* mutant as the template (Figure 6B). In the WT, a single band was amplified, and the gel showed the band to be approximately around 1 kbp. However, no amplification was observed in the *gly* mutant. When using the T-DNA-specific primer LBb1.3 in combination with RP, a specific band was amplified in the *gly* mutant, with a size ranging from 500 to 750 bp, consistent with the expected result. No such band was observed in the WT. These results confirm that the T-DNA insertion in the *AtGLYI3* gene of the *gly* mutant is homozygous at the DNA level.

To determine whether the *gly* mutant plants still expressed the *AtGLYI3* gene at the transcriptional level, RT-qPCR was performed. The results showed that (Figure 6C), compared to the WT, the expression level of *AtGLYI3* was significantly downregulated in the *gly* mutant. This indicates that the T-DNA insertion results in the loss of the transcriptional expression of *AtGLYI3*, confirming that the *gly* mutant is a functional null mutant.

#### 2.7.2. Impact of Homozygous Mutant on *A. thaliana* Self-Compatibility

**Figure 7:**
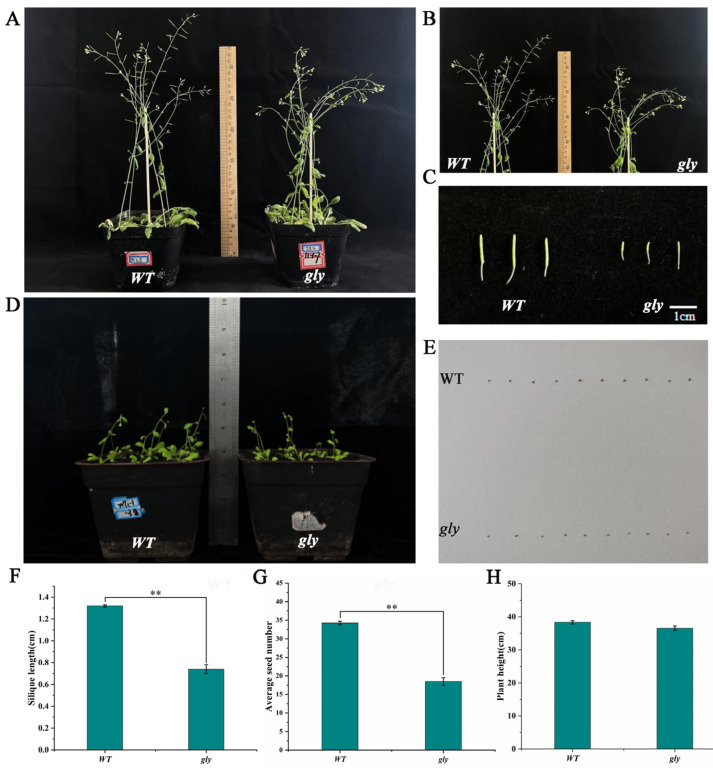
Phenotypes of WT and *gly*. (**A**) Plants with seeds germinated for 65 days. (**B**) Flower buds and siliques on the main stem. (**C**) Comparison of silique length and width between *gly* and WT plants. (**D**) Plants with seeds germinated for 28 days. (**E**) Comparison of seed size between *gly* and WT plants. (**F**) Silique lengths of *gly* and WT plants. The symbol “**” indicates that the mean expression value is significantly different from that of the WT (*p* < 0.01). (**G**) Average numbers of seeds in *gly* and WT plants. The symbol “**” indicates that the mean expression value is significantly different from that of the WT (*p* < 0.01). (**H**) Plant heights of *gly* and WT plants.

A phenotypic comparison was conducted by growing WT *A. thaliana* and the *gly* homozygous mutant plants together (Figure 7). The results showed that the mutant plants exhibited a significant reduction in their silique numbers, silique lengths, and seed numbers compared to the WT plants, indicating a clear decrease in SC. Additionally, the mutant plants produced significantly smaller seeds than the WT lines. While other traits, such as the plant height, were also reduced, the difference was not statistically significant. After 28 days of growth, no significant difference in the rosette leaf size was observed between the WT and *gly* mutant. These findings suggest that the mutation of this gene reduces SC in *A. thaliana* but does not affect the vegetative growth of the mutant plants. This indicates that the gene plays a role in the response to SC in *A. thaliana*.

## 3. Discussion

SI is a reproductive isolation mechanism in angiosperms that prevents inbreeding and helps maintain genetic diversity. In most plants, SI is controlled by the S locus, which includes stigma and pollen recognition genes that form different S haplotypes [25,26]. Based on genetic mechanisms, SI is classified into sporophytic self-incompatibility (SSI) and gametophytic self-incompatibility (GSI) [27]. GSI is common in Solanaceae and Rosaceae, involving mechanisms such as the S-nuclease system and SI-mediated programmed cell death, while SSI is primarily observed in the *Brassicaceae* family [28,29], where SI occurs between the pollen tube and the sporophytic papilla cells of the stigma. Pollen behavior in SSI is determined by the genotype of the diploid parent, a feature commonly observed in *Brassica* species, such as rapeseed and *A. thaliana* [30]. The pollination process in plants includes several stages: pollen adhesion, hydration, germination, pollen tube growth, and fertilization of the ovule. SI recognition can occur at any of these stages [31]. *B. rapa* is a self-incompatible crop, where self-incompatibility prevents self-pollination, making it challenging to maintain varietal purity and hindering crop improvement [32,33]. Therefore, understanding the mechanisms of self-compatibility in *B. rapa* is of significant importance.

MG is a cytotoxic metabolite produced during carbohydrate and lipid metabolism. Plants accumulate high concentrations of sugars during photosynthesis, which leads to the continuous production of MG in processes such as glycolysis and the Calvin cycle. Previous studies have shown that MG levels increase in response to various abiotic stresses. The glyoxalase system, consisting of GLYI and GLYII enzymes, converts MG into non-toxic d-lactate. Under stress conditions, the activity of these glyoxalase enzymes is upregulated to effectively reduce MG accumulation, thereby mitigating its potential damage and enhancing stress resistance in plants [13]. Over the past three decades, the comprehensive genome-wide identification of the *GLYI* and *GLYII* families has been conducted in species such as *A. thaliana*, rice, soybean, Chinese cabbage, and rapeseed. Studies have shown that *A. thaliana* and rice each contain 11 *GLYI* genes [11], soybean has 24 *GLYI* genes [12], Chinese cabbage has 15 *GLYI* homologous genes [13], and the *B. napus* genome contains 35 *GLYI* homologous genes [14].

Previous studies have demonstrated the involvement of GLY in various biological processes in plants [16], highlighting its critical role in enhancing plant resistance to abiotic stresses. However, as a multigene family, *GLY* may have additional, yet undiscovered roles and tissue-specific functions in plants. Sankaranarayanan et al. [8] showed that *GLYI* plays a key role in regulating the interaction between pollen and the pistil. Therefore, this study represents the first genome-wide identification of the *GLYI* family in *B. rapa*. By analyzing the members of the *BrGLYI* family, including their physicochemical properties, subcellular localization, and phylogenetic relationships, we found that many members of the *BrGLYI* gene family exhibit strong collinearity, with high intraspecific conservation. Promoter region analysis identified 31 cis-acting elements (Figure S3), suggesting that each gene member contains multiple cis-elements that regulate gene expression in response to various external stimuli. A comprehensive analysis of the *BrGLYI* family in *B. rapa* is expected to provide valuable insights into improving the plant’s stress resistance, self-compatibility, and pistil activity.

In conclusion, using various *B. rapa* cultivars, we performed a qPCR analysis that suggests that *BrGLYI13* plays a key role in the self-compatibility of *B. rapa*. This study uses *BrGLYI13* as a foundation for further exploration of its involvement in SC. We cloned an 852 bp gene sequence from the stigmas of *B. rapa* lines with different SC types, encoding a protein consisting of 283 amino acids. Multiple sequence alignments revealed no nucleotide mutations in the amino acid sequences. The BrGLYI13 protein sequence contains a highly conserved glyoxalase domain, confirming the high similarity and conservation of the gene. Predictive functional analysis indicated that it encodes a protein of 283 amino acids. Previous studies have shown that higher plants typically contain multiple members of the *GLYI*, *GLYII*, and *GLYIII* gene families, with differences in their subcellular localization, expression patterns, and functional roles. Sankaranarayanan et al. [18] reported that in *B. napus*, post-self-pollination, the ARC1-mediated degradation of GLO1 leads to increased MG levels, which is accompanied by an accumulation of MG-modified proteins (including GLO1). This accumulation targets and disrupts the papillary cells, ultimately resulting in pollen rejection. Building on this, we analyzed the expression levels of *BrGLYI13* in various tissues of *B. rapa*, including stigma tissues at different time points after self-pollination. We found that *BrGLYI13* is expressed in the leaves, stems, roots, and stigmas, with the highest expression observed in the stigma. Furthermore, the self-pollination process indicated that *BrGLYI13* plays a crucial role in regulating SC in *B. rapa*. These findings provide valuable insights for further investigation into the function of this gene in SC.

Although *A. thaliana* is inherently self-compatible, genetically modified self-incompatible *A. thaliana* plants have been successfully created by introducing SRK-SCR genes from closely related species. These findings suggest that *A. thaliana* possesses all the molecular components necessary for SI signaling, except for SRK and SCR. Due to its efficient transformation methods and abundant genetic resources, transgenic *A. thaliana* serves as a valuable model for evaluating the molecular components involved in the self-incompatibility mechanisms identified in *Brassica* species [34]

To investigate the function and potential mechanisms of the *BrGLYI13* gene in plant growth and development, we compared the phenotypes and expression levels of WT *A. thaliana* and a T-DNA insertion homozygous *gly* mutant. The results revealed significant phenotypic changes in the *gly* mutant compared to the WT, and these changes were stably inherited across subsequent generations. In the *gly* mutant, the expression of the *GLYI3* gene was markedly downregulated, suggesting that this mutant can be used for functional studies on *BrGLYI13*. However, the mechanisms by which the *AtGLYI3* gene influences the *A. thaliana* growth, development, and self-pollination metabolic pathways remain unclear and warrant further investigation. To further clarify the potential role of *BrGLYI13* in the SI pathway, future studies will focus on analyzing *BrGLYI13* overexpression lines and investigating variations in methylglyoxal (MG) levels. These experiments will help determine whether *BrGLYI13* directly or indirectly modulates the SRK/SCR signaling pathway.

Moreover, plant SI is not only a critical biological phenomenon but also holds significant agricultural value. Future research should delve deeper into the response mechanisms of the glyoxalase pathway in plant compatibility and leverage molecular approaches to breed more SI plant varieties.

## 4. Materials and Methods

### 4.1. Plant Materials and Growth Conditions

The experimental materials used were the self-compatible line 039-1 and the self-incompatible line GAU-28-5 of winter *B. rapa* (Table 2, Figure S4).

Seeds with full grains were selected and treated with 10% H_2_O_2_ for 30 min, followed by washing 2–3 times with sterile water. The seeds were then placed on Petri dishes with wet filter paper to germinate. After germination, the seeds were transplanted into pots (21 cm × 10 cm) and placed in a growth chamber under controlled conditions (25 °C with a 16 h light/8 h dark photoperiod) for further growth. When the seedlings reached the two-leaf stage, they were subjected to vernalization at 4 °C for 60 days before being transferred to a growth chamber at room temperature for continued growth. Upon flowering, flower buds that had similar growth patterns and were 1–2 days before flowering were selected. The flowers were emasculated before pollen dispersal. The unpollinated stigma, as well as stigmas collected 0, 5, 30, and 60 min after self-pollination, were collected, along with leaves, roots, stems, and flowers, and rapidly frozen in liquid nitrogen and stored at −80 °C for subsequent analysis. Each experiment was repeated three times.

The *A. thaliana* used in this experiment was the Col-0 ecotype (maintained in our laboratory for future use). Due to the high homology between *BrGLYI13* and *AtGLYI3* (*At1G11840*), we ordered the *AtGLYI3* T-DNA insertion mutant *gly* (SALK_103699.45.30.x) from AraShare Science (AraShare Technology Service Center, Fuzhou, China) to study the effect of *AtGLYI3* on the self-compatibility of *A. thaliana*, and the homozygous mutants identified through screening were used in the experiment. *A. thaliana* seeds were vernalized at 4 °C in the dark for 3 days and then disinfected three times with 75% ethanol, followed by 8 min of treatment with 15% sodium hypochlorite. After repeated washing with sterile water 8–10 times, the seeds were sown on solid 1/2 MS medium and grown at room temperature. When the seedlings developed 2–4 true leaves, they were transplanted into culture soil (peat/vermiculite/perlite = 3:1:1) and further grown under identical temperature and light conditions. After approximately four weeks, around 100 mg of *A. thaliana* leaves was quickly frozen in liquid nitrogen and stored at −80 °C for further analysis. Once mature, seeds from homozygous mutant plants were harvested, and after growing for 1–2 months, flowering pistils were quickly frozen in liquid nitrogen and stored at −80 °C for further analysis.

### 4.2. Genome-Wide Identification and Physicochemical Property Analysis of the BrGLYI Gene Family

The *A. thaliana* genome data were downloaded from the TAIR website (https://www.arabidopsis.org, accessed on 28 May 2024) [35]. The whole-genome sequence data of the *B. rapa* “Longyou 7” (NCBI, SRR18959686) [36] were obtained from previous sequencing conducted by the Rapeseed Laboratory of Gansu Agricultural University.

Based on the protein sequences of the 11 members of the *GLYI* gene family published in the model plant *A. thaliana* [11], BLAST alignment (E-value ≤ 10^−10^) was performed with the protein sequences of the whole genome of the “Longyou 7”. The glyoxalase (PF00903) domain of GLYI proteins was searched using the Pfam database(http://pfam-legacy.xfam.org/, accessed on 12 June 2024 ) by applying a hidden Markov model. The candidate genes identified from these two methods were submitted to the online tools SMART (https://www.Omicsclass.com/article/681, accessed on 12 June 2024) [37] and NCBI CDD (https://www.ncbi.nlm.nih.gov/cdd/, accessed on 12 June 2024) [38] for domain prediction. After removing redundant sequences, the final candidate sequences of the *GLYI* family members containing the conserved glyoxalase domain in *B. rapa* were obtained.

Upon acquiring the sequences, the amino acid composition, molecular weight, isoelectric point, hydrophilicity, and fat index of all family members were predicted using the online software ExPASy (https://web.expasy.org/protparam/, accessed on 15 June 2024) [39]. Additionally, the subcellular localization of *GLYI* family proteins was predicted using Plant-mPLoc (https://www.omicsclass.com/article/1438, accessed on 15 June 2024) [40].

### 4.3. Real-Time Quantitative Reverse Transcription PCR (RT-qPCR)

RNA was extracted from the roots, stems, leaves, self-pollinated and open-pollinated flowers, unpollinated stigmas, and stigmas at different time points after self-pollination from various self-compatible lines. The RNA was then reverse-transcribed into cDNA using a commercial kit (TIANGEN, Beijing, China). RT-qPCR was performed using the TIANGEN FastReal qPCR PreMix (SYBR Green) kit (TIANGEN, Beijing, China) in a 20 μL reaction system, containing both the target gene and reference gene, added to a 96-well plate. The RT-qPCR was carried out according to the manufacturer’s instructions, and the primers used for the RT-qPCR are listed in Table 3. Each reaction was carried out in triplicate. The relative expression levels of the genes were determined using the 2^−ΔΔCt^ method [41], with *Actin* as the internal control for the normalization of the target gene expression levels. Statistical significance was analyzed using SPSS 22.0, and data visualization was performed using Origin 2022.

### 4.4. Cloning of BrGLYI13

Gene cloning primers were designed with the assistance of Primer Premier 5.0 software: *GLYI13*-F: 5′-aatactagtggatccggtaccATGGCAGAAAATGCTGAGTTGTT-3′ and *GLYI13*-R: 5′-gcccttgctcaccatggtaccTTCCAGTTCCTTCAGAAAATCTTCA-3′. The cDNA extracted from unpollinated stigmas of the self-compatible line 039-1 and the self-incompatible line GAU-28-5, as well as from stigmas 30 min post-self-pollination, was used as templates for in vitro amplification by PCR under room temperature conditions. The detailed PCR reaction conditions were as follows: pre-denaturation at 94 °C for 2 min, followed by denaturation at 94 °C for 10 s, annealing at 60 °C for 30 s, and extension at 68 °C for 52 s, for a total of 35 cycles; ends were extended at 68 °C for 7 min and preservation at 4 °C. The amplified products were analyzed by 1% agarose gel electrophoresis, and the target bands were purified using the Agarose DNA Gel Recovery Kit (TIANGEN, Beijing, China). The purified PCR products were ligated into the pCAMBIAsuper1300-GFP vector using a ClonExpress II One Step Cloning Kit (Vazyme, Nanjing, China) and incubated at 25 °C for 18 h, followed by transformation into *E. coli* DH5α receptor cells. Positive recombinant clones (5 biological replicates) were selected through PCR and verified by sequencing. Primer synthesis and gene sequencing were performed by Sangon Biotech (Shanghai, China). Sequence alignment was performed using DNAMAN software.

### 4.5. Analysis of Cis-Regulatory Elements in the BrGLYI13 Gene Promoter

To further investigate the cis-regulatory elements within the promoter sequence, the 2000 bp upstream promoter sequence of the *BrGLYI13* gene was retrieved from the genome annotation file. The obtained sequence was then analyzed using the PlantCARE database, and the results were visualized using TBtools 2.154.

### 4.6. Identification and Phenotypic Observations of T-DNA Insertion Pure Mutants of A. thaliana AtGLYI3

#### 4.6.1. Identification of Homozygous Mutants at the DNA Level

A sufficient amount of Arabidopsis leaves was collected from −80 °C storage. Genomic DNA was extracted using the TIANGEN Plant Genomic DNA Extraction Kit (TIANGEN, Beijing, China), and the extracted DNA was used as a template for PCR amplification. The primer sequences for PCR were obtained from the SIGnAL database (http://signal.salk.edu/) (accessed on 12 March 2024) [42]. PCR amplification was performed using the gene-specific primers LP and RP and the T-DNA universal primer LB1.3. Homozygous *gly* mutants were selected, and the insertion site of the T-DNA was determined. Homozygous lines were identified and planted, and after approximately 3 weeks of growth, RT-qPCR was conducted to assess whether the *BrGLYI13* homologous gene *AtGLYI3* was silenced or downregulated in the mutants [43]. Phenotypic observations and analyses were then performed. A list of primers used is provided in Table 4.

The PCR reaction mixture (20 μL) consisted of 1 μL of template DNA, 1 μL of each forward and reverse primer, 10 μL of 2× Taq PCR MasterMix, and ddH_2_O to a final volume of 20 μL. After thorough mixing, PCR amplification was performed under the following conditions: initial denaturation at 94 °C for 3 min; 35 cycles of denaturation at 94 °C for 30 s, annealing at 58 °C for 30 s, and extension at 72 °C for 60 s; followed by a final extension at 72 °C for 5 min, with preservation at 4 °C. PCR products were analyzed by 1.2% agarose gel electrophoresis, and the results were visualized using a gel imaging system.

#### 4.6.2. RT-qPCR Verification of *AtGLYI3* Expression in Homozygous Mutants

Based on the results of the expression localization analysis, flowering stigmas from homozygous *gly* mutants and WT *A. thaliana* were selected at an appropriate growth stage for further investigation. Total RNA was extracted and then reverse-transcribed into cDNA and was subsequently analyzed using RT-qPCR to assess gene expression levels. *Actin2* (*AT3G18780*) was used as the internal control for the normalization of the target gene expression levels. The analysis method was the same as that described in Section 4.3. The primers employed for the RT-qPCR are provided in Table 4.

#### 4.6.3. Phenotypic Observation of Homozygous Mutant Plants

Ten WT *A. thaliana* and ten homozygous *gly* mutant plants were randomly selected. The phenotypic traits, such as the silique length and average seed number, were observed and recorded.

## 5. Conclusions

This study identified 17 *BrGLYI* genes in *B. rapa*, which were classified into six distinct groups based on their phylogenetic relationships. Notably, *BrGLYI13* exhibited significant specificity across different varieties and tissues, with particularly high expression in the flowers of the self-compatible lines. The cloned *BrGLYI13* gene encodes a protein of 283 amino acids, which contains a conserved glyoxalase domain. Promoter analysis revealed that this gene is associated with cis-acting elements linked to light, hormones, and stress responses. Subcellular localization prediction indicated that the protein is expressed in the cytoplasm. Expression analysis showed that *BrGLYI13* is highly expressed in the pistils, suggesting its potential role in regulating pistil activity. Phenotypic analysis of Arabidopsis mutant lines revealed that the *gly* mutant exhibited significantly reduced silique lengths and seed numbers compared to the WT, which suggests that the *AtGLYI3* gene is essential for the process of self-pollination. Based on these findings, it is speculated that *BrGLYI13* shares a similar function with *AtGLYI3*. In conclusion, *BrGLYI13* appears to be a key regulatory factor in self-pollination compatibility in *B. rapa*, playing an important role in regulating MG levels during self-pollination. This study lays the groundwork for further research on SC in *B. rapa* and offers valuable insights for breeding strategies aimed at enhancing crop yields and improving SC.

## Figures and Tables

**Table 1:** The information on *BrGLYI* gene family in winter *B. rapa*.

Gene Name	Sequence ID	Number of Amino Acids /aa	Molecular Weight /Da	pI	Instability Index	Aliphatic Index	Grand Average of Hydropathicity	Subcellular Localization Prediction
*BrGLYI1*	*Brapa02T002698.1*	167	18,791.65	5.81	62.94	89.22	−0.193	Cytoplasm
*BrGLYI2*	*Brapa03T001219.1*	193	21,797.96	8.43	37.75	90.41	−0.461	Chloroplast
*BrGLYI3*	*Brapa04T002215.1*	185	13,878.76	6.08	42.68	85.61	−0.601	Nucleus
*BrGLYI4*	*Brapa05T001273.1*	137	15,252.22	5.46	58.99	76.86	−0.226	Cytoplasm
*BrGLYI5*	*Brapa06T000491.1*	171	19,274.70	7.77	36.81	78.60	−0.405	Cytoplasm
*BrGLYI6*	*Brapa06T000534.1*	235	26,352.75	6.32	32.14	69.74	−0.460	Cytoplasm
*BrGLYI7*	*Brapa06T000824.1*	1044	115,660.97	8.27	44.05	82.90	−0.298	Cytoplasm
*BrGLYI8*	*Brapa06T001102.1*	174	19,778.79	5.68	65.91	89.54	−0.209	Nucleus
*BrGLYI9*	*Brapa06T001420.1*	266	30,367.20	5.24	43.82	70.38	−0.575	Cytoplasm
*BrGLYI10*	*Brapa07T002043.1*	185	21,015.55	4.84	37.80	73.78	−0.709	Cytoplasm
*BrGLYI11*	*Brapa07T003470.1*	406	45,190.31	8.85	34.98	89.09	−0.111	Chloroplast
*BrGLYI12*	*Brapa08T003039.1*	167	19,784.93	5.88	49.09	95.69	−0.217	Cytoplasm
*BrGLYI13*	*Brapa08T003191.1*	283	31,886.44	5.27	28.95	82.61	−0.322	Cytoplasm
*BrGLYI14*	*Brapa09T000323.1*	137	15,394.22	5.84	33.70	71.09	−0.464	Cytoplasm
*BrGLYI15*	*Brapa09T000758.1*	172	19,692.81	6.30	48.25	87.79	−0.252	Cytoplasm
*BrGLYI16*	*Brapa09T005172.1*	118	12,967.91	6.40	45.49	100.76	0.069	Nucleus
*BrGLYI17*	*Brapa10T001850.1*	195	21,959.00	6.71	43.16	89.54	−0.449	Chloroplast

**Table 2:** Source and compatibility of experimental materials.

Variety (Line)	Source	Compatibility	Relative Compatibility Index
039-1	Gansu Agricultural University	Self-compatibility	1.00
GAU-28-5	Gansu Agricultural University	Self-incompatibility	0.07

Note: The relative compatibility index standards are as follows: 0.01–0.1 indicates high incompatibility, 0.1–0.2 indicates incompatibility, 0.2–0.4 indicates compatibility, and 0.4–1.0 indicates high compatibility. The relative compatibility index is calculated as the average number of self-pollinated siliques divided by the average number of cross-pollinated plants.

**Table 3:** RT-qPCR primer sequences.

Gene Name	Forward Primer Sequence (5′–3′)	Reverse Primer Sequence (5′–3′)
*BrGLYI1*	TGAGAGCAGTGGTGGAAGAAGG	AAGAGGCACGACGGGAAGG
*BrGLYI2*	AGATTCTGGATAAAGCTGGGATTGC	TTGTGTCTGGATCACGAGTGAATATC
*BrGLYI3*	CGTTAAACCATGTCTCAAGACTTTGC	ACGCTCTGTCTCCACGAATCC
*BrGLYI4*	ACTCCTTCCTTCGCTCTCTCAAG	GCTACCTCTAATCCGTTTCCATCAG
*BrGLYI5*	ATGGTGCGGTGGCTGTGAG	GTGGCTTCCGATGCGTACAAC
*BrGLYI6*	CCACTGCTCTCGGTCGGATC	GCTGCTGATTCCTTCGCTTCC
*BrGLYI7*	AGGATGAGGAGGAGAAGAAGAAGAAG	AAGTAGCGGCGTGACAGAGG
*BrGLYI8*	GGACATGGAATTGGAATACATCTCTTG	TGACATACTCTCGCACTGGAAGG
*BrGLYI9*	GAAGGAGAAGCAGTGGCGATTC	CGAGGGTTAAGGATGGGATGGG
*BrGLYI10*	CGGTGCGTGGCTATTCAACTAC	TGTGATTATCCATCGGGTCCAAATG
*BrGLYI11*	GTTATGCTCCGTGTTGGTGATCTC	AACTTGTACTCTGGATTGTCTCTTGTG
*BrGLYI12*	AGTATGGGAGCAGTGGAGAAGAAG	ATCAGGGTCGTGGAAGAAGAGC
*BrGLYI13*	CTGAAGTAGTGAAGATAGCCAACCAAG	GGTGCCGAGTCCAGGAAGAG
*BrGLYI14*	TCAGCGAGCCAGAGGACAAG	ACCAATGCGGACAACGATGC
*BrGLYI15*	GGAATTGGAATACATCTCTTGCGTTC	TCTCCACTGCTGCCATACTCTC
*BrGLYI16*	AGCTTCAGTCTGGTCCTCTCAAG	GTGAACGACAGTAGCGAAGAGTATC
*BrGLYI17*	GCGAGAACTTAGAACGGTCACTG	TACCCATAACCATGCTCCTCTATACG
*Actin*	TGTGCCAATCTACGAGGGTTT	TTTCCCGCTCTGCTGTTGT

**Table 4:** Primer sequences for RT-qPCR.

Gene Name	Sequence (5′~3′)
*gly*-LP	TGTAACGCGTCTGTGATGATC
*gly*-RP	CCCAAAGCCAGTTCCAATATC
LBb1.3	ATTTTGCCGATTTCGGAAC
*AtGLYI3*-F	CAGTCAAAGGTGGAGGCAGTGTC
*AtGLYI3*-R	TTGGCAGAAAGGTTCAGGAGTTGG
*Actin2*-F	GTTGGGATGAACCAGAAGGA
*Actin2*-R	GCTCTTCAGGAGCAATACGAAG

## Data Availability

The original contributions presented in this study are included in the article/Supplementary Materials. Further inquiries can be directed to the corresponding author(s).

## Supplementary Materials

The following supporting information can be downloaded at https://www.mdpi.com/article/10.3390/ijms26062737/s1.

## References

[1] Ye X.Y., Qiu X.M., Wang Y., Li Z.G. (2019). Glyoxalase system and its role in response and adaptation of plants to environmental stress. Plant Physiol. J..

[2] Yadav S.K., Singla-Pareek S.L., Ray M., Reddy M.K., Sopory S.K. (2005). Methylglyoxal levels in plants under salinity stress are dependent on glyoxalase I and glutathione. Biochem. Biophys. Res. Commun..

[3] Parvin K., Hasanuzzaman M., Bhuyan M., Mohsin S.M., Fujita A.M. (2019). Quercetin Mediated Salt Tolerance in Tomato through the Enhancement of Plant Antioxidant Defense and Glyoxalase Systems. Plants.

[4] Welchen E., Schmitz J., Fuchs P., García L., Wagner S., Wienstroer J., Schertl P., Braun H.P., Schwarzländer M., Gonzalez D.H. (2016). D-Lactate Dehydrogenase Links Methylglyoxal Degradation and Electron Transport through Cytochrome c. Plant Physiol..

[5] Li Z.G. (2016). Methylglyoxal and Glyoxalase System in Plants: Old Players, New Concepts. Bot. Rev..

[6] Klebanoff S.J. (1958). The effect of X-radiation on the glutathione metabolism of intact erythrocytes in vitro. J. Gen. Physiol..

[7] Klebanoff S.J. (1958). The relationship of hydrogen peroxide to the inhibition of the glyoxalase activity of intact erythrocytes by X-radiation. J. Gen. Physiol..

[8] Sankaranarayanan S., Jamshed M., Kumar A., Skori L., Scandola S., Wang T., Spiegel D., Samuel M.A. (2017). Glyoxalase Goes Green: The Expanding Roles of Glyoxalase in Plants. Int. J. Mol. Sci..

[9] Guo Y.L. (2013). Gene family evolution in green plants with emphasis on the origination and evolution of *Arabidopsis thaliana* genes. Plant J..

[10] Xu M., Zuo D., Wang Q., Lv L., Zhang Y., Jiao H., Zhang X., Yang Y., Song G., Cheng H. (2023). Identification and molecular evolution of the GLX genes in 21 plant species: A focus on the Gossypium hirsutum. BMC Genom..

[11] Mustafiz A., Singh A.K., Pareek A., Sopory S.K., Singla-Pareek S.L. (2011). Genome-wide analysis of rice and *Arabidopsis* identifies two glyoxalase genes that are highly expressed in abiotic stresses. Funct. Integr. Genom..

[12] Ghosh A., Islam T. (2016). Genome-wide analysis and expression profiling of glyoxalase gene families in soybean (*Glycine max*) indicate their development and abiotic stress specific response. BMC Plant Biol..

[13] Yan G., Xiao X., Wang N., Zhang F., Gao G., Xu K., Chen B., Qiao J., Wu X. (2018). Genome-wide analysis and expression profiles of glyoxalase gene families in Chinese cabbage (*Brassica rapa* L.). PLoS ONE.

[14] Yan G., Zhang M., Guan W., Zhang F., Dai W., Yuan L., Gao G., Xu K., Chen B., Li L. (2023). Genome-Wide Identification and Functional Characterization of Stress Related Glyoxalase Genes in *Brassica napus* L. Int. J. Mol. Sci..

[15] Singla-Pareek S.L., Kaur C., Kumar B., Pareek A., Sopory S.K. (2020). Reassessing plant glyoxalases: Large family and expanding functions. New Phytol..

[16] Kaur C., Singla-Pareek S.L., Sopory S.K. (2014). Glyoxalase and Methylglyoxal as Biomarkers for Plant Stress Tolerance. Crit. Rev. Plant Sci..

[17] Schmitz J., Dittmar I.C., Brockmann J.D., Schmidt M., Hüdig M., Rossoni A.W., Maurino V.G. (2017). Defense against Reactive Carbonyl Species Involves at Least Three Subcellular Compartments Where Individual Components of the System Respond to Cellular Sugar Status. Plant Cell.

[18] Sankaranarayanan S., Jamshed M., Samuel M.A. (2015). Degradation of glyoxalase I in *Brassica napus* stigma leads to self-incompatibility response. Nat. Plants.

[19] Takayama S., Shimosato H., Shiba H., Funato M., Che F.S., Watanabe M., Iwano M., Isogai A. (2001). Direct ligand-receptor complex interaction controls Brassica self-incompatibility. Nature.

[20] Dixit R., Nasrallah J.B. (2001). Recognizing self in the self-incompatibility response. Plant Physiol..

[21] Krogh A., Larsson B., von Heijne G., Sonnhammer E.L. (2001). Predicting transmembrane protein topology with a hidden Markov model: Application to complete genomes. J. Mol. Biol..

[22] Sun W.C., Ma W.G., Lei J.M., Liu Q., Yang R.Y., Wu J.Y., Wang X.F., Ye J., Zeng J., Zhang Y.H. (2007). Study on Adaptation and Introduction Possibility of Winter Rapeseed to Dry and Cold Areas in Northwest China. Sci. Agric. Sin..

[23] Liu H.Q., Sun W.C., Liu Z.G., Wang Z.J., Fang Y., Wu J.Y., Li X.C., Fang Y. (2015). Safe wintering and economic and ecological benefit of winter rapeseed in dry and cold areas of northern China. Ying Yong Sheng Tai Xue Bao.

[24] Luo Y.X. (2007). Study on Self-incompatibility and Pollinations of *Brassica compestris* L. Acta Agric. Boreali-Occident..

[25] Zhang Y., Xue Y., Franklin-Tong V.E. (2008). Molecular Biology of S-Rnase-Based Self-Incompatibility. Self-Incompatibility in Flowering Plants: Evolution, Diversity, and Mechanisms.

[26] Sassa H. (2016). Molecular mechanism of the S-RNase-based gametophytic self-incompatibility in fruit trees of Rosaceae. Breed. Sci..

[27] Takayama S., Isogai A. (2005). Self-incompatibility in plants. Annu. Rev. Plant Biol..

[28] Zhao H., Zhang Y., Zhang H., Song Y., Zhao F., Zhang Y., Zhu S., Zhang H., Zhou Z., Guo H. (2022). Origin, loss, and regain of self-incompatibility in angiosperms. Plant Cell.

[29] Zhang T., Wang K., Dou S., Gao E., Hussey P.J., Lin Z., Wang P. (2024). Exo84c-regulated degradation is involved in the normal self-incompatible response in Brassicaceae. Cell Rep..

[30] Hiscock S.J., McInnis S.M. (2003). Pollen recognition and rejection during the sporophytic self-incompatibility response: Brassica and beyond. Trends Plant Sci..

[31] Zhang Y., Zhao Z., Xue Y. (2009). Roles of proteolysis in plant self-incompatibility. Annu. Rev. Plant Biol..

[32] He Y.T., Cheng B.Y., Fu T.D., Li D.R., Tu J.X. (2003). Origins and Evolution of *Brassica campestris* L. in China. J. Genet. Genom..

[33] Lei J.M., Wu J.Y., Zhang Y. Analysis on the self-compatibility of winter rapeseed (*Brassica rapa*) in China. Proceedings of the 12th International Rapeseed Congress.

[34] Yamamoto M., Nishio T. (2014). Commonalities and differences between Brassica and Arabidopsis self-incompatibility. Hortic. Res..

[35] Lamesch P., Berardini T.Z., Li D., Swarbreck D., Wilks C., Sasidharan R., Muller R., Dreher K., Alexander D.L., Garcia-Hernandez M. (2012). The Arabidopsis Information Resource (TAIR): Improved gene annotation and new tools. Nucleic Acids Res..

[36] Wu J., Xu X.-D., Liu L.J., Ma L., Pu Y., Wang W., Hua X.-Y., Song J.-M., Liu K., Lu G. (2022). A Chromosome Level Genome Assembly of a Winter Turnip Rape (*Brassica rapa* L.) to Explore the Genetic Basis of Cold Tolerance. Front. Plant Sci..

[37] Letunic I., Bork P. (2018). 20 Years of the SMART protein domain annotation resource. Nucleic Acids Res..

[38] Yang M., Derbyshire M.K., Yamashita R.A., Marchler-Bauer A. (2020). NCBI’s Conserved Domain Database and Tools for Protein Domain Analysis. Curr. Protoc. Bioinform..

[39] Wilkins M.R., Gasteiger E., Bairoch A., Sanchez J.C., Williams K.L., Appel R.D., Hochstrasser D.F. (1999). Protein identification and analysis tools in the ExPASy server. Methods Mol. Biol..

[40] Chou K.C., Shen H.B. (2010). Plant-mPLoc: A top-down strategy to augment the power for predicting plant protein subcellular localization. PLoS ONE.

[41] Livak K.J., Schmittgen T.D. (2001). Analysis of relative gene expression data using real-time quantitative PCR and the 2^−ΔΔCT^ Method. Methods.

[42] Liang X., Peng L., Baek C.H., Katzen F. (2013). Single step BP/LR combined Gateway reactions. Biotechniques.

[43] Xiong H., Lü G., Li D.B., Li S.S. (2020). Molecular Identification and Phenotype of *Arabidopsis thaliana* Mutant erd15. J. Trop. Subtrop. Bot..

