# Supplementary material for: Cloning and Functional Analysis of Glyoxalase I Gene BrGLYI 13 in Brassica rapa L
Source: Int J Mol Sci. 2025 Mar 18;26(6):2737. doi: 10.3390/ijms26062737 (PMC11942965; doi:10.3390/ijms26062737)
Supplement: Supplementary file 1 [file ijms-26-02737-s001.zip › ijms-3501804-supplementary.pdf]

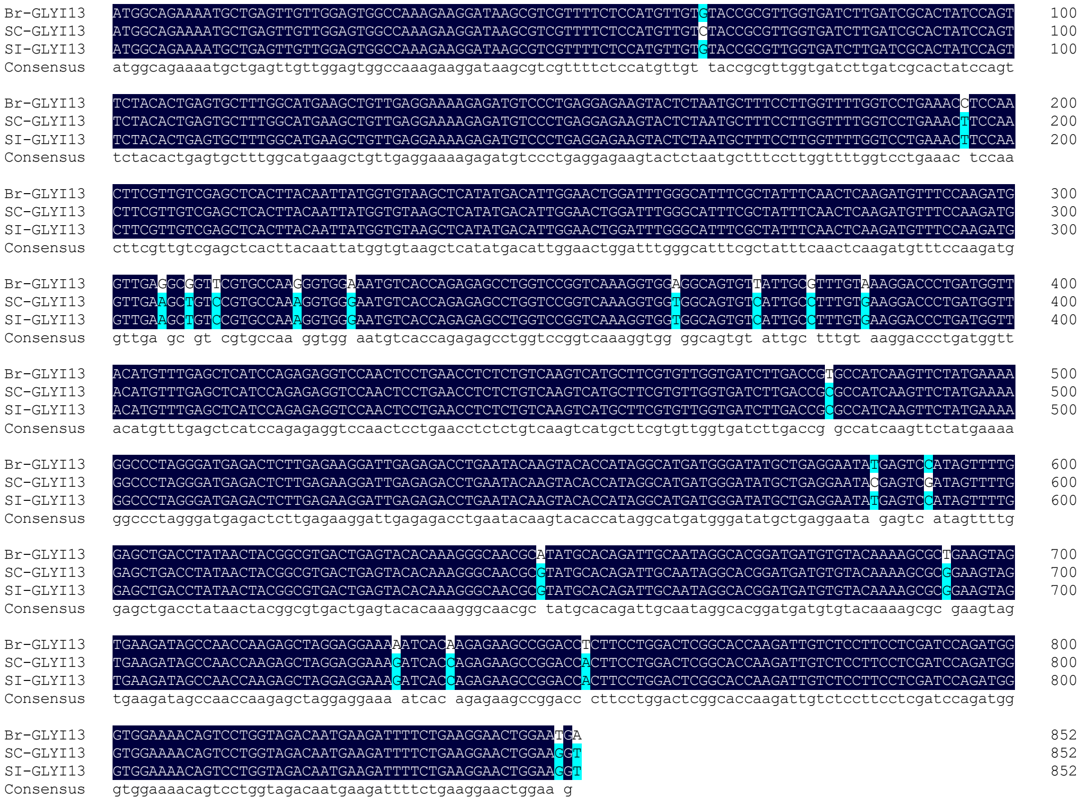

Figure. S1 Results of *BrGLYI13* DNAs sequence alignment, with a consistency of 97.54%.

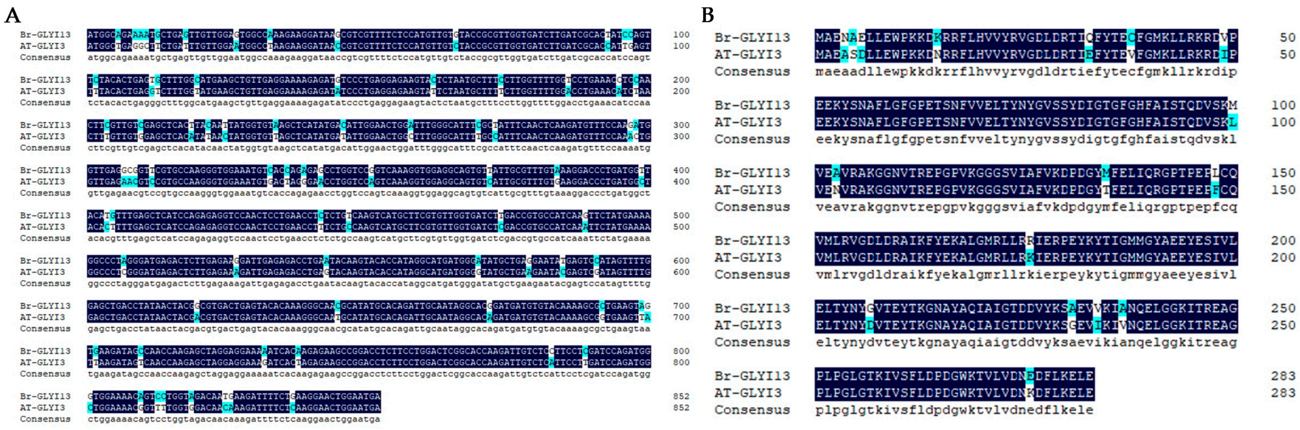

Figure. S2 The sequence alignment results of homologous genes between winter Brassica rapa and Arabidopsis thaliana. (A) DNA sequence alignment, with a consistency of 90.50%. (B) Protein sequence alignment, with a consistency of 93.66%.

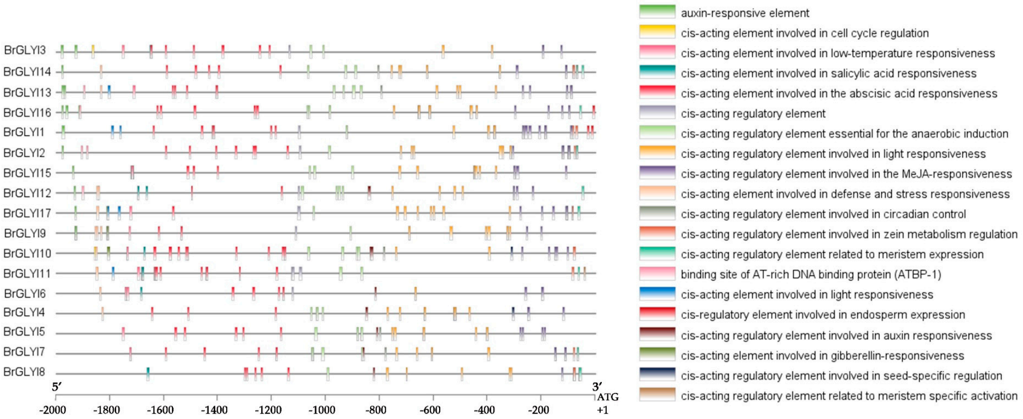

Figure. S3 Analysis of cis-acting elements of *BrGLYI* gene family members. Different colors represent different cis-acting elements. Note: +1 is the "A" of the initiating ATG codon.

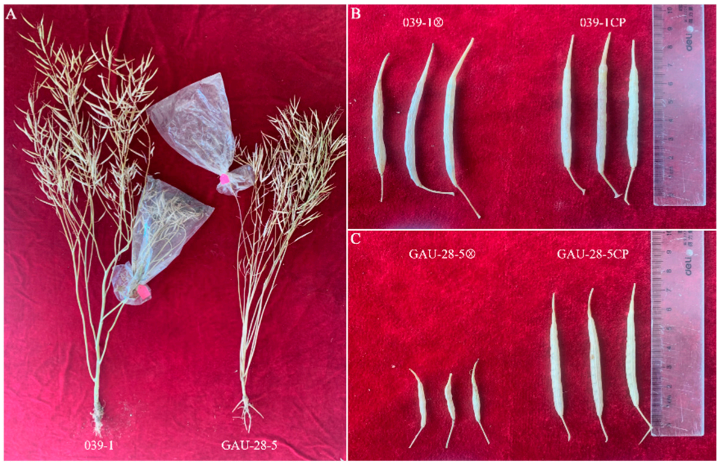

Figure. S4 Field plant phenotypes of different varieties (lines): (A) Comparison of plant phenotypes of different compatibility lines and quantity and size of corner silique in bagging; (B) The left is 039-1 self-pollinated silique phenotype, and the right is 039-1 cross-pollinated silique phenotype; (C) GAU-28-5 self-pollinated silique phenotype on the left and GAU-28-5 cross-pollinated silique phenotype on the right. ⊗ indicates self-pollinated, CP indicates cross-pollinated.

AtGLYI sequences:

>AtGLYI1

MAANMMRPAFAYTVVYVKDVAKSVEFYSRAFGHNVRRLDESHRWGELESGQTTIA FTPLHQHETDDLTGKVQATQSARERAPIEVCFCYPDVDAAFKRAVENGAEAVSKPEDKEW

GQKVGYVRDIDGIVVRIGSHVK

>AtGLYI2

MSSYSIASAISRISPLIRFVKPYSTGFSFITCACNSTRRPKRFDQLCVFSMASEARESPA NNPGLSTNRDEATKGYIMQQTMFRIKDPKASLDFYSRVLGMSLLKRLDFSEMKFSLYFLG YEDTTTAPTDPTERTVWTFGQPATIELTHNWGTESDPEFKGYHNGNSEPRGFGHIGVTVDD VHKACERFEELGVEFAKKPNDGKMKNIAFIKDPDGYWIEIFDLKTIGTTTVNAA

>AtGLYI3

MNEIASASMLRLCQCFISICNVHFVSMRAAESSFLLSRNMAEASDLLEWPKKDNRRF LHVVYRVGDLDRTIEFYTEVFGMKLLRKRDIPEEKYSNAFLGFGPETSNFVVELTYNYGVS SYDIGTGFGHFAISTQDVSKLVENVRAKGGNVTREPGPVKGGGSVIAFVKDPDGYTFELIQ RGPTPEPFCQVMLRVGDLDRAIKFYEKALGMRLLRKIERPEYKYTIGMMGYAEEYESIVL ELTYNYDVTEYTKGNAYAQIAIGTDDVYKSGEVIKIVNQELGGKITREAGPLPGLGTKIVS FLDPDGWKTVLVDNKDFLKELE

>AtGLYI4

MKEDAGNPLHLTSLNHVSVLCRSVDESMNFYQKVLGFIPIRRPESLNFEGAWLFGHG IGIHLLCAPEPEKLPKKTAINPKDNHISFQCESMGVVEKKLEEMGIDYVRALVEEGGIQVD QLFFHDPDGFMIEICNCDSLPVVPLVGEMARSCSRVKLHQMVQPQPQTQIHQVVYP

>AtGLYI5

MATASFRWILQLHRDVPKAARFYEKGLDFSVNVVTLRWAELQSGPLKLALMQAPSE HVMSEKGYSSLLSFTVADINTTISKLMELGAELDGSIKYEVHGKVASVRCLDGHVLGLYEP S

>AtGLYI6

MVRIIPMAASSIRPSLACFSDSPRFPISLLSRNLSRTLHVPQSQLFGLTSHKLLRRSVNC LGVAESGKAAQATTQDDLLTWVKNDKRRMLHVVYRVGDMDRTIKFYTECLGMKLLRKR DIPEEKYTNAFLGYGPEDSHFVIELTYNYGVDKYDIGAGFGHFGIAVDDVAKTVELVKAK GGKVSREPGPVKGGKTVIAFIEDPDGYKFELLERGPTPEPLCQVMLRVGDLDRAIKFYEK AFGMELLRTRDNPEYKYTIAMMGYGPEDKFPVLELTYNYGVTEYDKGNAYAQIAIGTDD VYKTAEAIKLFGGKITREPGPLPGISTKITACLDPDGWKSVFVDNIDFLKELE

>AtGLYI7

MKDETGNPLHIKSLNHISLLCRSVEESISFYQNVLGFLPIRRPDSFDFDGAWLFGHGIGI

HLLQSPEPEKLLKKTEINPKDNHISFQCESMEAVEKKLKEMEIEYVRAVVEEGGIQVDQLF
FHDPDAFMIEICNCDSLPVIPLAGEMARSCSRLNIRQLVQPTQIHP

>AtGLYI8

MEEKKKKGDDELNSKPPLMALNHVSRLCKDVKKSLEFYTKVLGFVEIERPASFDFD
GAWLFNYGVGIHLVQAKDQDKLPSDTDHLDPMDNHISFQCEDMEALEKRLKEVKVKYIK
RTVGDEKDAAIDQLFFNDPDGFMVEICNCENLELVPCHSADAIRLPEDRHAPPVALPDSSN
RRMPQPNS

>AtGLYI9

MASLGHIARESSDITRLAQFYKEVFGFEEIESPDFGDLQVVWLNLPGAFAMHIIQRNP
STNLPEGPYSATSAVKDPSHLPMGHHICFSVPNFDSFLHSLKEKGIETFQKSLPDGKVKQVF
FFDPDGNGLEVASRS

>AtGLYI10

MATASFRWILQLHRDVPKAARFYAQGLDFSVNVVTLRWAELHSGPIKLALMQSPSNH
VAEKGYSSLLSFTVTDINTTVTKLMALGAELDGTIKYEIHGKVAAMKCPDGYMLGLYEAA

>AtGLYI11

MASIFRPSSASLDLRPKVICTNLSTKERFEFQKKSVRKERINVRFYSLKAKAQGSSIEG
ISVVQEKELNNKTDYGVVGVHHVGLLCENLERSLEFYQNILGLEINEARPHDKLPYRGAW
LWVGSEMIHLMELPNPDPLTGRPEHGGRDRHACIAIRDVSNLKEILDKAGIAYTMSKSGRP
AIFTRDPDANALEFTQV

BnaGLYI **sequences：**

>BnaGLYI1

MKENTGNPLHLKSLNHISLLCRSVEESMNFYQHVLGFLPIRRPGSFDFDGAWLFGHG
VGIHLLQSTEPEKLLKKTEINPKDNHISFQCESMGAVEKKLKEMGIEYVRAVVEEGGIQVD
QLFFHDPDGFMIEICNCDSLPVVPLAGEMARSCSRVNIHQLVQPPQIHP*

>BnaGLYI2

MASIFRPSISLDLRPKVTCTNLSTKERLDFHNKSLRKEKLNIRLRGVKANQAQGTSVV
TEEKELNNKTDYGVVGVHHVGLLCENLERSLEFYQNTLGLEINEARPHDKLPYRGAWLW

VGSEMIHLMELPNPDPLTGRPEHGGRDRHACIAIRDVSVLKEILDKAGIAYTMSKSGRPAIF
TRDPDTNALEFTQV*

>BnaGLYI3

MATLGHIARESSDVTRLALFYKEVFGFEEIESPDFGDLKVIWLNLPGAFAMHIIQRNTS
TNLPEGPYSATSAVRDPSHLPMGHHICFSVSNFDSFLRSLKEKGIETFQKSLPDGKVKQVFF
FDPDGNGLEVASRSES*

>BnaGLYI4

MASNVIKPAYAYTVVYVRDVAKSVEFYSRAFGYNVRRLDESHRWGELESGQTTIAFT
PLHQHETDDLTGKVQSSTRSERERAPLEVCFCYADVDAAFKRAVENGAVAVSEPEDKEWG
QKVGYVRDIDGIVVRIGSHVKSYKPWTSFGFSLAINLNLPVVPQLNNITSKKRQ*

>BnaGLYI5

MGSYSIASAISRVSVSPLNRFVKLYSTAFSYINCPCNRALRPKRFDQLRVFSMASEAKE
SAANNPGLSTVRDEATKGYIMQQTMFRVKDPKASLDFYSRVLGMSLLKRLDFSEMKFSLY
FLGYEDTSTAPTDPTERTVWTFGRPATIELTHNWGTESDPEFKGYHNGNSEPRGFGHIGVT
VDDVHKACERFEQLGVEFVKKPNDGKMKNIAFIKDPDGYWIEIFDLKTIGTTAGNAA*

>BnaGLYI6

MALRCFPIWACPQTAYYHYPLLGFDIKRRRIPLWECSSSASQRAVTAVGSEVPYGREL
KKPSDEMGLTQESPQLETFHRDLSMLPKPLTANSLTSSAGDDSKVRISFQGIPGAYSETAAL
KAYPNCETVPCDQFETAFQAVELWLVDKAVLPIENSVGGSIHRNYDLLLRHRLHIVQEVHL
PVNHCLLGVPGVSIEDIKCVLSHPQALDQCVNSLNDLGIQRVSAKDTATAAQTVSSSGERSI
GAVASVRAANIYGLDILAENIQDDANNVTRFLILARDPMIPRTDRPYKTSIVFSLEEGPGVL
FKALAVFSLRNINLSKIESRPQRRRPLRVVDGSNNGCAKYFDYLFYIDFEASMAETRAQHA
LGHLQEFTSFIRILGCYPMDLVNSSLTILSSSKLSFKLFIGETNIITFFLNIFGGVINFESNEKR
KTYIKINKFFNMEIGRRVLRQIKMSKTQICLCFIIFLYIWSSGSSREVVGQLETDCFSSDGRS
KSSGICSPRASSLRKLADVASLDWPKNDTRRFFHVVYRVGDLDRTIKFYTECFGMKVSRQ
RDVPKEKYSNAFMGFGSEKSHFAVELTYNYGVSSYDIGDGFGHFTISTQDVYKMVETVRA
KGGNVTREPGPVEGGSSIIAIVKDPDGYPFELIQRGPTPEPFCQVMLRVGDLDRAIKFYEKA
LGMRLLRRIKKPEYKYTIGMMGFNESVVLELTYKYGVTEYKKGNAYAQIAIGTDDVYKS
GEVVKIVNKELGGKITREPGPLPGIGTKIVSFLDPDGWKTVLVDNKDFMKELGESSVNYV
SSSVKNLEEAPEAKTKSQSFEEEEEKKKMRGGSLWQLGQSITRRLAQSDKKPLSRRYLAS

GADLKKTALYDFHVAHGGKMVPFSGWSMPIQYKDSIIDSTVNCRVNGSLFDVAHMCGLS
LKGKDCVPFLEKLVVADVAGLAPGTGSLTVFTNEKGGAIDDSVITKVTDEHIYLVVNDGC
RDKDLAHIEEHMKAFKSKGGPLAAPLLQHLTKEDLSKLYFGQFQILDINGSTCFLTRTSSR
GRVQEDLTVNESLCFGFTAKAILEKSEGKVRLTGLGARDSLRLEAGLSLYGNDMEQHISPV
EAGLTWAIGKRRRAEGGFLGADVILKQLEDEPTIRRVRFFSSGPPARSHSEVHDENGNKIG
EITSGGFSPNLKKNIAMGYVKSGQHKTGTKVKILVRGKPYEGNITKMPFVANKYYKPS*

>BnaGLYI7

MKENAGNPLHLTSLNHVSLLCRSIEESMVFYQTVLGFFPIRRPESLNFEGAWLFGHGI
GIHLLRSSEPEKLPKKTEINPKDNHISFQCESMSAVEKKLEEMEIEYVRAIVEEGGIQVDQL
FFHDPDGFMIEICNCDSLPVVPLIGGMARSCSRVKLHQMVQPQQQTQIHQVVHP*

>BnaGLYI8

MEQKNKSDESRPPLMALNHVSRLCRDVKKSLEFYTKVLGFVETERPASLDFDGAWL
FNYGVGIHLVQAKDEEKLPSNTDHLDPMDNHISFQCEDMEALEKRLKEVDVKYIKRTVG
EQEDAAIDQLFFNDPDGFMVEICNCENLELKPRDSADAIRLPGDRHAPPVSLPGSSDHADD
TRLPQTNS*

>BnaGLYI9

MRIISTASTIRPSLLGCVSASSPRFPVVSRNLSFSHVTQSKLLTLRRSVSCLGVAESGKA
STAATEEDLLKWVKDDNRRMLHVVYRVGDLDRTIKFYTECLGMKLLRKRDIPEEKYTNA
FLGYGPEDSHFVIELTYNYGVDKYDIGAGFGHFGIAVDDVAKTVELIKAKGGKVTREPGA
VKGGKTVIAFIEDPDGYKFELLERGPTPEPLCQVMLRVGDLDRSIKFYEKAFGMELLRTRD
NPEYKYTIAMMGYGPEDKTAVLELTYNYGVTEYDKGNAYAQIAIGTNDVYKTAEAVKLF
GGKITREPGPLPGISTKITACLDPDGWKSVFVDNVDFLKELE*

>BnaGLYI10

MKENAGNPLHLTSLNHVSLLCRSIEESMNFYQRVLGFFPIRRPESLNFEGAWLFGHGI
GIHLLRALELEKLPKKNEINPKDNHISFQCESMGAVEKKLDEMEIDYVRSKVEEGGIQVDQ
LFFHDPDGFMIEICNCDSLPIVPLVGGMVRSCSRVKLHQMVQPQPQIQINQVVHP*

>BnaGLYI11

MAENADLLEWPKKDKRRFLHVVYRVGDLDRTIQFYTECFGMKLLRKRDVPEEKYS
NAFLGFGPETSNFVVELTYNYGVSSYDIGTGFGHFAISTQDVSKMVEAVRAKGGNVTREP
GPVKGGGSVIAFVKDPDGYMFELIQRGPTPEPLCQVMLRVGDLDRAIKFYEKALGMRLLR

RIERPEYKYTIGMMGYAEEYESIVLELTYNYGVTEYTKGNAYAQIAIGTDDVYKSAEVVKI
ANQELGGKITREAGPLPGLGTKIVSFLDPDGWKTVLVDNEDFLKELE*

>BnaGLYI12

MASNIMRPAFAYTVVYVKDVAKSVEFYSRAFGHNVRRLDESHRWGELESGQTTIAFT
PRHQHETDDLTGKVQATHSDPERAPIEVCFCYPDVDAAFKRAVENGAVAVSEPEDKEWGQ
KVGYVRDIDGIVVRIGSHVK*

>BnaGLYI13

MGHENAAVSESQHHDDAASAASPGFKLVGFSKFVRKNPKSDKFKVKRFHHIEFWCG
DATNVARRFSWGLGMRFSAKSDLSTGNMVHASYLLTSGDLRFLFTAPYSPSLSAGETSTAS
IPSFDHVSCRSFFSSHGLGVRAVAIEVEDAESAFSISVANGAVPSSPPNVLNGAVTIAEVKLY
GDVVLRYVSYHNGAVNFLPGFESVDDTSSFPLDYGIRRLDHAVGNVPELGPALTYLAGFT
GFHQFAEFTADDVGTAESGLNSAVLANNDEMVLLPVNEPVHGTKRKSQIQTFLEHNEGAG
LQHLALMSEDIFRTLREMRKRSGVGGFDFMPSPPPTYYKNLKKRIGDVLSDEQIRECEELG
ILVDRDDQGTLLQIFTKPLGDRPTIFIEIIQRVGCMKKDEEGKVYQSGGCGGFGKGNFSELF
KSIEEYEKTLEAKQLVG*

>BnaGLYI14

MKENAVNPLRLTSLNHVSLLCRSLEESMNFYQKVLGFFPVRRPESLDFEGAWLFGHG
IGIHLLRSTEPEKLPKKTAINSKDNHISFQCESMAAVEKKLDEMEIEYVREIVEGRGIKVDQI
FFHDPDGFMIEICNCDSLPVVPLVGGLAQYCAKVKLHQMGQPQPQTN*

>BnaGLYI15

MGHENAAVSENQHHDDDEAAATSASPGFKLVGFSKFVRKNPKSDKFKVKRFHHIEF
WCGDATNVARRFSWGLGMRFSAKSDLSTGNMVHASYLLTSGDLRFLFTAPYSPSLSAGE
NPPTTTASIPSFDHVTYRSFFSSHGLGVRAVAVEVEDAESAFSISVSNGAVPSSPPIVLNDAV
TIAEVKLYGDVVLRYVSYKVATVFLPRFETVDDTSSFPLDYGIRRLDHAVGNVPELGPALT
YLSRLTGFHQFAEFTADDVGTAESGLNSAVLANNDETVLLPVNEPVHGTKRKSQIQTYLE
HNEGAGVQHLALMSEDIFRTLREMRKRSGVGGFDFMPSPPPTYYKNLKNRVGDVLSEEQI
EECEELGILVDRDDQGTLLQIFTKPLGDRPTIFIEIIQRIGCMKKDEEGRVYQSGGCGGFGK
GNFSELFKSIEEYEKTLEAKQLVG*

>BnaGLYI16

MASIFRPSVSLDLRPKVSCTNHLPAIERFEFQKNKNLRKDRLHGSLKANQAQGSAEGI

IVVQEKEINNQTDYGVVGVHHVGLLCENLERSLEFYQNILGLEINEARPHDKLPYRGAWL
WVGSEMIHLMELPNPDPLTGRPEHGGRDRHACIAIRDVSNLKAILDKAGIEYTMSRSGRPA
IFTRDPDANALEFTQV*

>BnaGLYI17

MKENTGNPLHLKSLNHISLLCRSVEESMNFYQHVLGFLPIRRPGSFDFDGAWLFGHG
VGIHLLQSTEPEKLLKKTEINPKDNHISFQCESMGAVEKKLNEMGIEYVRAVGEEGGIQVD
QLFFHDPDGFMIEICNCDSLPVVPLAGEMARSCSRVNIHQLVQPPQIHP*

>BnaGLYI18

MKENTGNPLHLKSLNHISLLCRSVEESMNFYQHVLGFLPIRRPGSFDFDGAWLFGHG
VGIHLLQSTEPEKLLKKTEINPKDNHISFQCESMGAVEKKLNEMGIEYVRAVVEEGGIQVD
QLFFHDPDGFMIEICNCDSLPVVPLAGEMARSCSRVNIHQLVQPPQIHP*

>BnaGLYI19

MASIFRPSISLDLRPKVTCTNLSTKERLEFHNKSLRKEKLNIGLRGVKANQAQGTSVV
TEEKELNNKTDYGVVGVHHVGLLCENLERSLEFYQNILGLEINEARPHDKLPYRGAWLW
VGSEMIHLMELPNPDPLTGRPEHGGRDRHACIAIRDVSVLKEILDKAGIAYTMSKSGRPAIF
TRDPDTNALEFTQV*

>BnaGLYI20

MPSFLSFVKSPGETTRSMFDLSRLLKRLDFSEMKFSLYFLGYEDTSTAPTERTVWTFG
RPATIELTHNWVTESDPEFKGGNSEPREFGNTGVTVDDVHKACERFEQLGVEFVKKPNDG
KMKNIAFIKDPDGYWIEIFNLKSIGTTTGNAA*

>BnaGLYI21

MGHENAAVSENQHHDGDAAATSASPGFKLVGFSKFVRKNPKSDKFKVKRFHHIEFW
CGDATNVARRFSWGLGMRFSSKSDLSTGNMVHASYLLTSGDLRFLFTAPFSPSISAGEIPPN
TTASIPSFDHVTYHSFFSSHGLGVRAVAIEVEDAESAFSVSISNGAVPSSPPIALNDAVTIAEV
KLYGDVVLRYVSYKAVTSVFLPRFETVEDTSSFPLDYGIRRLDHAVGNVPELGPALTYLSR
FTGFHQFAEFTADDVGTAESGLNSAVLANNDETVLLPVNEPVHGTKRKSQIQTYLEHNEG
AGVQHLALMSEDIFKTLREMRKRSGVGGFDFMPSPPPTYYKNLKNRVGDVLSDEEIKECE
ELGILVDRDDQGTLLQIFTKPLGDRPTIFIEIIQRIGCMKKDEEGRVYQSGGCGGFGKGNFS
ELFKSIEEYEKTLEAKQLVG*

>BnaGLYI22

MASNIIKPAYAYTVVYVRDVAKSVEFYSRAFGYNVRRLDESHRWGELESGQTTIAFTP LHQHETDDLTGKVQSSTRSDRERAPLEVCFCYHDVDAAFKRAVENGAVAVSEPEDKEWG QKVGYVRDIDGIVVRIGSHVK*

>BnaGLYI23

MGSYSIASAISRFAPLTRFVKPYSTASSLISCPWNRAQRPKRFDKLRVFSMASEAKESP ANNPGLSTVRDEATKGYIMQQTMFRVKDPKASLDFYSRVLGMSLLKRLDFSEMKFSLYFL GYEDTSTAPTDPTERTVWTFGRPATIELTHNWGTESDPEFKGYHNGNSEPRGFGHIGVTVD DVHKACERFEQLGVEFVKKPNDGKMKNIAFIKDPDGYWIEIFDLKTIGTTAGNAA*

>BnaGLYI24

MALRCFPIWVCPQTAYYHYPLLGFDTKRRRICLWECSSSASQRAITAVGGDVPYGREL KKPSDEMGLTQERPQLETFHRDLSMLPKPLTANSLTSSAWDDSKVRISFQGIPGAYSETAAL KAYPNCETVPCDQFETAFQAVELWLVDKAVLPIENSVGGSIHRNYDLLLRHRLHIAQEVHL PVNHCLLGVPGVQKEDIKCVLSHPQALDQCVNSLNELGIQRVSAKDTATAAQTVSSSGER SIGAVASVRAANIYGLDILAQNIQDDANNVTRFLILARDPMIPRTDRPYKTSIVFSLEEGPG VLFKALAVFALRNINLSKIESRPQRGRPLRVVDGSNNGCAKYFDYLFYIDFEASMAETCAQ HALGHLQEFTSFIRILGCYPMDLDSSLTILSSSKLSFKLFIAETNSSSREVVGQLESDCFSSD GRSKSSGICSPRASYLRKLADVASNGELLDWPKNDTRRFFHVVYRVGDLDRTIKFYTECF GMKVSRQRDVPKEKYSNAFMGFGSEKSHFAIMVLAHMKLKMDLGISPFQLKMQVYKM VETVRAKGGNVTREPGPVEGGSSIIAIVKDPDGYPFELIQRGPTPEPFCQVMLRVGDLDGA IKFYEKALGMRLLRRIEKPEYKYTIGMMGYNESVVLELAYNYGVTEYKKGNAYAQIAIGT DDVYKSGEVVKIVNKELGGDITREPGPLPGIDTKIVSFLDPDGWKTVLVDNKDFMKELG*

>BnaGLYI25

MKENAGNPLHLTSLNHVSLLCRSIEESMVFYQTVLGFFPIRRPESLNFEGAWLFGHGI GIHLLRSSEPEKLPKKTEINPKDNHISFQCESMSAVEKKLEEMEIEYVRAIVEEGGIQVDQL FFHDPDGFMIEICNCDSLPVVPLVGGMARSCSRVKLHQMVQPQPQTQIHQVVHP*

>BnaGLYI26

MRIISMASTIRPSLLGCVSASPRFPVVSRNLSRSTLSFSHVTQSKLLTLRRSVSCLGVAE SGNASTAATEEDLLKWVKDDNRRMLHVVYRVGDLDRTIKFYTECLGMKLLRKRDIPEEK YTNAFLGYGPEDSHFVIELTYNYGVDKYDIGAGFGHFGIAVDDVAKTVELIKAKGGKVTR EPGAVKGGKTVIAFIEDPDGYKFELLERGPTPEPLCQVMLRVGDLDRSIKFYEKAFGMELL

RTRDNPEYKYTIAMMGYGPEDKTAVLELTYNYGVTEYDKGNAYAQIAIGTDDVYKTAEAVKLFGGKITREPGPLPGISTKITACLDPDGWKSVFVDNVDFLKELE*

>BnaGLYI27

MAENADLVEWPKKDKRRFLHVVYRVGDLDRTIQFYTECFGMKVLRKRDVPEEKYSNAFLGFGPETSNFVVELTYNYGVSSYDIGTGFGHFAISTQDVSKMVEAVRAKGGNVTREPGPVKGGGSVIAFVKDPDGYTFELIQRGPTPEPLCQVMLRVGDLDRAIKFYEKALGMRLLRRIERPEYKYTIGMMGYAEEYESIVLELTYNYGVTEYTKGNAYAQIAIGTDDVYKSAEVVKIANQELGGKITREAGPLPGLGTKIVSFLDPDGWKTVLVDNEDFLKELE*

>BnaGLYI28

MKENPGNPLHLTSLNHVSLLCRSIEESMNFYRKVLGFFPIRRPESLNFEGAWLFGHGIGIHLLRALEPEKLSTKNEINPKDNHISFQCESMGAVEKKLEEMEIDYVRSKVEEGGIQVDQLFFHDPDGFMIEICNCDSLPIVPLVGGMVRSCSRVKLHQMVQPQPQTQINQVVHP*

>BnaGLYI29

MSFLGKMKENAVNPLRLTSLNHVSLLCRSLEESMNFYQKVLGFFPVRRPESLDFEGAWLFGHGIGIHLLGSTEPEKLPKKTAINSKDNHISFQCESMAAVEKKLDEMEIEYVREIVEGRGIKVDQIFFHDPDGFMIEICNCDSLPVVPLVGGLAQYYAKVKLHQMGQPQPQTN*

>BnaGLYI30

MGHENAAVCETQQHDDAASPGFKLVGFSKFVRKNPKSDKFKVKRFHHIEFWCGDATNVSRRFSWGLGMRFSAKSDLSTGNMVHASYLLTSGDLRFLFTAPYSPSLSAGEAQPSATASIPSFDHASCRSFFSSHGLGVRAVAIEVEDAESAFSISVANGAVPSSPPNVLNGAVTIAEVKLYGDVVLRYVSYYNGTVSFLPGFESVDDTSSFPLDYGIRRLDHAVGNVPELGPALTYLAGFTGFHQFAEFTADDVGTAESGLNSAVLANNDEMVLLPINEPVHGTKRKSQIQTFLEHNEGAGLQHLALMSEDIFRTLREMRKRSGVGGFDFMPSPPPTYYKNLKKRVGDVLSEEQIEECEELGILVDRDDQGTLLQIFTKPLGDRPTIFIEIIQRVGCMKRDEEGKVYQSGGCGGFGKGNFSELFKSIEEYEKTLEAKQLVG*

>BnaGLYI31

MLQLRIRLVDAIYYGVVGVHHVGLLCKNLERSLEFYQNILGLEINEARPHDKLPYRGAWLWVGSEMIHLMELPNPDPLTGRPEHGGRDRHACIAIRDVSNLKKILEHFSSFPHFLPSCFCFTKTQNLMLSGF*

>BnaGLYI32

MASNIMRPAFAYTVVYVKDVAKSVEFYSRAFGHNVRRLDESHRWGELESGQTTIAFT
PRHQHETDDLTGKVQATRSDRERTPIEVCFCYPDVDAAFKRAVENGAVAVSEPEDKEWGQ
KVGYVRDIDGIVVRIGSHVK*

>BnaGLYI33

MFRVKDPKASLDFYSRVLGMSLLKRLDFSEMKFSLYFLVKIFSDTSTAPTDPTATIELT
HNWGTESDPEFKGGNSEPREFGHTGVTVDDVHKACERFEQLEVEFVKKPNDGKMKNIAF
IKDPDGYWIEIFNLKSIGTTTGNAA*

>BnaGLYI34

MIVNGFQIPPSHAESVSRIDAGFKLDWLEKKLDIMFQVKDPKASLDFYSRVLGMSLL
KRLDFSEMKFSLYFLGYEDTSTAPTDPTERLNQIQANGIVEAQFLCMTNY*

>BnaGLYI35

MATLGHIARESSDVTRLAQFYKEVFGFEEIESPDFGDLKVIWLNLPGAFAMHIIQRNT
STNLPEGPYSATSAVRDPSHLPMGHHICFSVSNFDSFLRSLKEKGIDTFQKSLPDGKVKQVF
FFDPDGNGLEVASRSES*

>lcl|KJ755985.1_cds_AIE90139.1_1

ATGGCAGAAAATGCTGATTTGGTGGAGTGGCCAAAGAAGGATAAGCGTCGTTTTC
TCCATGTTGTGTACCGCGTTGGTGATCTTGATCGCACTATCCAGTTTTACACAGAGTGCT
TTGGCATGAAGGTGTTGAGGAAAAGAGATGTCCCTGAGGAGAAGTACTCTAATGCCTT
CCTTGGGTTTGGTCCTGAAACCTCCAACTTCGTTGTCGAGCTCACTTACAATTATGGTG
TTAGCTCATATGACATTGGAACTGGATTTGGGCATTTCGCTATTTCAACTCAAGATGTTT
CTAAGATGGTTGAGGCGGTTCGTGCCAAGGGTGGAAATGTCACCAGAGAGCCTGGTC
CGGTCAAAGGTGGAGGCAGTGTCATTGCCTTTGTCAAGGACCCTGATGGTTACACTTT
TGAGCTTATCCAGAGAGGTCCAACTCCTGAACCTCTCTGTCAAGTCATGCTTCGTGTTG
GTGATCTTGACCGCGCCATCAAGTTCTATGAAAAGGCCCTTGGGATGAGACTCTTGAG
AAGGATTGAGAGGCCTGAATACAAGTACACCATAGGCATGATGGGATATGCTGAGGAA
TATGAGTCAATAGTTTTGGAGCTGACCTATAACTACGGTGTGACTGAGTACACAAAGGG
CAACGCATATGCACAGATTGCAATAGGCACGGATGATGTGTACAAAAGCGCTGAAGTA
GTGAAGATAGCCAACCAAGAGCTAGGAGGAAAGATCACTAGAGAAGCCGGACCTCTT
CCTGGACTCGGCACCAAGATTGCCTCATTCCTCGATCCAGATGGCTGGAAAACAGTTC

TGGTAGACAACGAAGATTTTCTCAAGGAACTGGAATGA

>Brapa08T003191.1 upstream 2000 bp promoter sequence

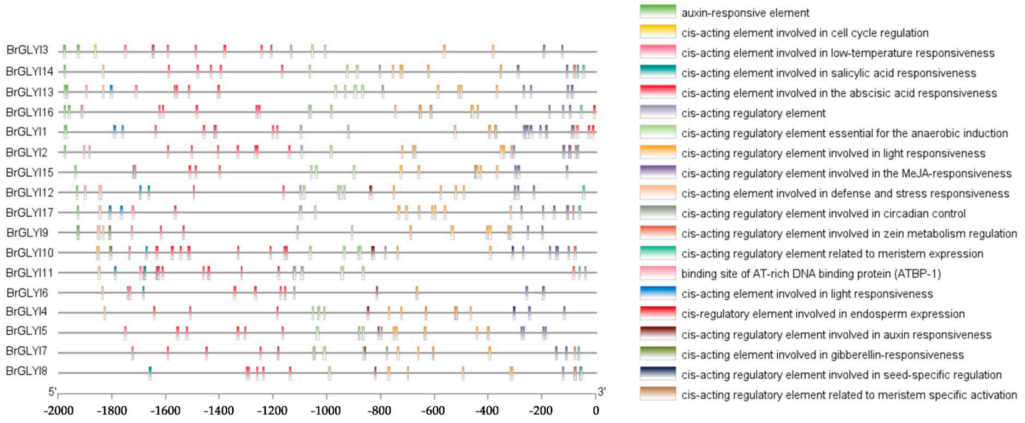

agattgaagattacaatgaagaagaggaagaagagaagaggagggacggtggaagacatgctagtgaatggcattgaattgattctgaaactcttctggttcggattcagactcaggtacagatccttgcattatcttcctgctctttcgattgatttatttagtgtttggttgagataagagaagagagatttagacataatgttagttaaggggtttagtactgctaaaagatttatgtctctctctctatctgtgtttcacgtgaatacactctgctcgctgctcctctcccccacacacaaactcgttgaatttcagacagagttaacttacgataatagccttcaatagtgataaaaagtgccagctacacatcactgtatttcactatatttcgagtgcataaaagtattattcaaaatactaaattttcgtacacgaaagtattattcaaaataaaaagttttcgtaccatattatagtattcaagtgaattcattcaaaattgaaaaactttccataagagtgttttatactttatatatatatcaaaaagttcatttgtatttttggttattaattagtttctagtattgtctttcaatgttggtgttgaccgcatgcgattaaaaagtgggaaaataaaagaagattataatgtgccctttaggtatgagttttctctaatctctctattattcagttattattgaaaatatccatcgaaagcattgaaatacagtctagactctagagaaataaagacaggaatacaacaactccattcttttgttttgttgagtagataaaaaatacattgactagaagaggcctattgtatttattttgttgatgataagctgataacacatttcctcctctcaaccaataaactaacttaataaatgcttttagaaaccaaaactattgcgacaagttgctgcaggtaactaacaaatgtaacgttccacatacagtatatcataacaatctagggaacatagtgctgcattagaaagaaaaaaaattataaaacagcaagccgacaaaacttgtttatcaattatcagagaggtccatctggcaactcttttgatatataaaagctatcaaacagaagaagaggacagcttaatttctagagaagttaaattcaaaacatttgttataagattctgaccatgtgtcttctgttcatatgccatatgcatccaaacattactatcgtcgagattttaaaaatatactaaaatactaaaatatactaatatactaaaatatactaaatattttagttatttttataacatgcatcatgcatgtagatgtaaaaaatgtaaattgaaaacaaactaatcaaacaaaaaaattgcatataacgcaacattcatatacttcaagtagtgggcttttctccttcatatgtatgggcttacacccatgtgccttgggcttttatctcatcataataatacacttcattattgtactatctgccgacaaaaaaaaaatcgtttctgtacgaccacgtgtatcccatgggtattaggttgacatcccatcgttccccaaacaactaatctaacggctgtaatacgtctgtgatggttaaactacatcacaggcagtatccgagaagttgtcactcacttagttcttttttataagtacagtgacggactttgctttctatctacagaaaactatcaaagcccagtgctgtgtgtatcttctatcaggtgagtaactgtcgcagatgatctcttttctacatttcgatgaatcgtatcctcgttgatcgcactctagatctcatttgcttgtcatatgaaacattcagaatcatatattttgattgaatctgggttaattgtggatcatcgttcgattgatcttatgggtttcatgggatctcattgttctgtgttacggtgatgatgatcattgagaaaggtgtttaaagtttg

gatttttatctcttttatgtagaaaa

BrGLYI sequences:

>BrGLYI1

MKENTGNPLHLKSLNHISLLCRSVEESMSFYQHVLGFLPIRRPGSFDFDGAWLFGHG VGIHLLQSTEPEKLLKKTEINPKDNHISFQCESMGAVEKKLKEMGIEYVRAVVEEGGIQVD QLFFHDPDGFMIEICNCDSLPVVPLAGEMARSCSRVNIHQLVQPPQIHP*

>BrGLYI2

MEQKKNKSDESRPPLMALNHVSRLCRDVKKSLEFYTKVLGFVETERPASLDFDGAW LFNYGVGIHLVQAKDEEKLPSNTDHLDPMDNHISFQCEDMEALEKRLKEVDVKYIKRTV GEQEDAAIDQLFFNDPDGFMVEICNCENLELKPRDSADAIRLPGDRHAPPVSLPGSSDHAD DTRLPQTNS*

>BrGLYI3

MDPRELIGRHAYIVLGRGQGADIRVVTDTATALGRIIHRTSRFDQLRVFSMASEAKES AANNPGLSTVRDEATKGYIMQQTMFRVKDPKASLDFYSRVLGMSLLKRLDFSEMKFSLYF LGYEDTSTAPTDPTERTVWTFGRPATIELTHNWGTESDPEFKGYHNGNSEPRGFGHIGVTV DDVHKACERFEQLGVEFVKKPNDGKMKNIAFIKDPDGYWIEIFDLKTIGTTAGNAA*

>BrGLYI4

MKENAGNPLHLTSLNHVSLLCRSIEESMVFYQTVLGFFPIRRPESLNFEGAWLFGHGI GIHLLRSSEPEKLPKKTEINPKDNHISFQCESMSAVEKKLEEMEIEYVRAIVEEGGIQVDQL FFHDPDGFMIEICNCDSLPVVPLIGGMARSCSRVKLHQMVQPQQQTQIHQVVHP*

>BrGLYI5

MATASFRWILQLHKDVPKAARFYAQGLDFSVNVVTLRWAELQSGPLKLALMQSPSD HVVSEKGYSSLLSFTVTDINTSISKLMELGAELDGSIKYEVHGKVASVRCLDGHVLGLYEP S*

>BrGLYI6

MSFLKMKENAVNPLRLTSLNHVSLLCRSLEESMNFYQKVLGFFRVRRPESLDFEGAW LFGHGIGIHLLRSTEPEKLPKKTAINSKDNHISFQCESMAAVEKKLDEMEIEYVREIVEGRG IKVDQIFFHDPDGFMIEICNWDSLPVVPLVGGLAQYCAKVKLHQMGQPQPQTN*

>BrGLYI7

MASNIMRPAFAYTVVYVKDVAKSVEFYSRAFGHNVRRLDESHRWGELESGQTTIAFT
PRHQHETDDLTGKVQATHSDPERAPIEVCFCYPDVDAAFKRAVENGAVAVSEPEDKEWGQ
KVGYVRDIDGIVVRIGSHVK*

>BrGLYI8

MATLGHIARESSDVTRLALFYKEVFGFEEIESPDFGDLKVIWLNLPGAFAMHIIQRNTS
TNLPEGPYSATSAVRDPSHLPMGHHICFSVSNFDSFLRSLKEKGIETFQKSLPDGKVKQVFF
FDPDGNGLEVASRSES*

>BrGLYI9

MALRCFPIWACPQTAYYHYPLLGFDIKRRRIPLWECSSSASQRAVTAVGSEVPYGREL
KKPSDEMGLTQESPQLETFHRDLSMLPKPLTANSLTSSAGDDSKVRISFQGIPGAYSETAAL
KAYPNCETVPCDQFETAFQAVELWLVDKAVLPIENSVGGSIHRNYDLLLRHRLHIVQEVHL
PVNHCLLGVPGVSIEDIKCVLSHPQALDQCVNSLNDLGIQRVSAKDTATAAQTVSSSGERSI
GAVASVRAANIYGLDILAENIQDDANNVTRFLILARDPMIPRTDRPYKTSIVFSLEEGPGVL
FKALAVFSLRNINLSKIESRPQRRRPLRVVDGSNNGCAKYFDYLFYIDFEASMAETRAQHA
LGHLQEFTSFIRILGCYPMDLVSSSREVVGQLETDCFSSDGRSKSSGICSPRASSLRKLADV
ASNGELLDWPKNDTRRFFHVVYRVGDLDRTIKFYTECFGMKVSRQRDVPEEKYSNAFMG
FGSEKSHFAVELTYNYGVSSYDIGDGFGHFTISTQDVCGNVTREPGPVEGGSSIIAIVKDPD
GYPFELIQRGPTPEPFCQVMLRVGDLDRAIKFYEKALGMRLLRRIKKPEYKYTIGMMGFN
ESVVLELTYKYGVTEYKKGNAYAQIAIGTDDVYKSGEVVKIVNKELGGKITREPGPLPGIG
TKIVSFLDPDGWKTVLVDNKDFMKELGESSVNYVSSSVKNLEEAPEAKTKSQSFEDEEEK
KKKMRGGSLWQLGQSITRRLAQSDKKPLSRRYLASGADLKKTALYDFHVAHGGKMVPFS
GWSMPIQYKDSIIDSTVNCRVNGSLFDVAHMCGLSLKGKDCVPFLEKLVVADVAGLAPGT
GSLTVFTNEKGGAIDDSVITKVTDEHIYLVVNDGCRDKDLAHIEEHMKAFKSKGGDVLW
HIHDERSLLALQGPLAAPLLQHLTKEDLSKLYFGQFQILDINGSTCFLTRTRRVGFFSSGPPA
RSHSEVHDENGNKIGEITSGGFSPNLKKNIAMGYVKSGQHKTGTKVKILVRGKPYEGNIT
KMPFVATKYYKPS*

>BrGLYI10

MRIISTASTIRPSLLGCVSASSPRFPVVSRNLSFSHVTQSKLLTLRRSVSCLGVAESGKA
STAATEEDLLKWVKDDNRRMLHVVYRVGDLDRTIKFYTECLGMKLLRKRDIPEEKYTNA

FLGYGPEDSHFVIELTYNYGVDKYDIGAGFGHFGIAVDDVAKTVELIKAKGGKVTREPGA
VKGGKTVIAFIEDPDGYKFELLERGPTPEPLCQVMLRVGDLDRSIKFYEKAFGMELLRTRD
NPEYKFLRANPTKLVRLIGLVWFICLKSLAVSDCLQYTIAMMGYGPEDKTAVLELTYNYG
VTEYDKGNAYAQIAIGTDDVYKTAEAVKLFGGKITREPGPLPGISTKITACLDPDGWKCLW
TTLIFSKNWSERRKEATRMASFIAMKATGGWVVCLTSAGVSYIYL*

>BrGLYI11

MASIFRPSVSLDLRPKVSCTNHLPAIERFEFQKNKNLRKDRLHGSLKANQAQGSAEGI
IVVQEKEINNQTDYGVVGVHHVGLLCENLERSLEFYQNILGLEINEARPHDKLPYRGAWL
WVGSEMIHLMELPNPDPLTGRPEHGGRDRHACIAIRDVSNLKAILDKAGIEYTMSRSGRPA
IFTRDPDANALEFTQV*

>BrGLYI12

MTTRMAGFGEEESGISDSEIIEGFLRPKIEGEAVAIPGLIVELGEELYTREPWLLPQTAH
PILNPREWLYLGKPNRNNFPVEGVDHEGCWVRIGSAILIRSEETDEIIGGTSRFRYRFRNKG
DKHALRWSNWFMKELLKRLDFSEMKFSFYFLGYEDTSTDPTDPTERTVWTFGRPATIELT
HNWGTESDPEFKGGNSEPREFGHTGVTVDDVHKACKRLEQLGVEFVKKPNDGKMKNIA
FIKDPDGYWIEIFNLKSIGTTTGNAA*

>BrGLYI13

MAENAELLEWPKKDKRRFLHVVYRVGDLDRTIQFYTECFGMKLLRKRDVPEEKYSN
AFLGFGPETSNFVVELTYNYGVSSYDIGTGFGHFAISTQDVSKMVEAVRAKGGNVTREPG
PVKGGGSVIAFVKDPDGYMFELIQRGPTPEPLCQVMLRVGDLDRAIKFYEKALGMRLLRR
IERPEYKYTIGMMGYAEEYESIVLELTYNYGVTEYTKGNAYAQIAIGTDDVYKSAEVVKIA
NQELGGKITREAGPLPGLGTKIVSFLDPDGWKTVLVDNEDFLKELE*

>BrGLYI14

MASNVIKPAYAYTVVYVRDVAKSVEFYSRAFGYNVRRLDESHRWGELESGQTTIAFT
PLHQHETDDLTGKVQSSTRSERERAPLEVCFCYADVDAAFKRAVENGAVAVSEPEDKEWG
QKVGYVRDIDGIVVRIGSHVKSYKPWTSFGFSLAINLNLPVVPQLNNITSKKRQ*

>BrGLYI15

MASIFRPSISLDLRPKVTCTNLSTKERLDFHNKSLRKEKLNIRLRGVKANQAQGTSVV
TEEKELNNKTDYGVVGVHHVGLLCENLERSLEFYQNILGLEINEARPHDKLPYRGAWLW
VGSEMIHLMELPNPDPLTGRPEHGGRDRHACIAIRDVSVLKEILDKAGIAYTMSKSGRPAIF

TRDPDTNALEFTQV*

>BrGLYI16

MKENAGNPLHLTSLNHVSLLCRSIEESMNFYQKVLGFFPIRRPESLNLEGAWLFGHGI
GIHLLRALELEKLPKKNEINPKDNHISFQCESMGAVEKKLDEMEIDYVRSKVEEGGIQVDQ
LFFHDPDGFMIEICNCDSLPIVPLVGGMVRSCSRVKLHQMVQPQPQIQINQVVHP*

>BrGLYI17

MEKNESRPPLMALNHVSRLCIDVKKSLEFYTKVLGFVETERPASLDFSGAVGIHLVQV
KDEEKLPSSNTDHLEKRLKEICNCENLERNSADAIHLPGDRRAPSVAIPGRLDREDANRLP
QTNF*
